# Precise Electrocatalysis on Fe‐Porphyrin Conjugated Networks Achieves Energy‐Efficient Extraction of Uranium

**DOI:** 10.1002/advs.202409084

**Published:** 2024-10-07

**Authors:** Wenwen Wang, Meiyun Xu, Haotian Wu, Yan Song, Peng Liu, Haisheng Yu, Linjuan Zhang, Shusen Chen, Daoben Hua

**Affiliations:** ^1^ State Key Laboratory of Radiation Medicine and Protection School for Radiological and Interdisciplinary Sciences (RAD−X) Collaborative Innovation Center of Radiological Medicine of Jiangsu Higher Education Institutions Soochow University Suzhou 215123 China; ^2^ Beijing Research Institute of Chemical Engineering and Metallurgy CNNC Key Laboratory on Uranium Extraction from Seawater China National Nuclear Corporation Beijing 101149 China; ^3^ Key Laboratory of Interfacial Physics and Technology Shanghai Institute of Applied Physics Chinese Academy of Sciences Shanghai 201800 China

**Keywords:** electrocatalysis, faraday efficiency, Fe‐porphyrin conjugated network, pre‐storing electrons, uranium extraction

## Abstract

Electrochemical extraction has the potential to enhance uranium (U) extraction capacity and rates, but thus far, high selectivity and energy efficiency have not been achieved through the design of electrode materials. Herein, a precise electrocatalysis strategy is developed using a Ferrum (Fe) porphyrin‐phenanthroline conjugated network (Fe@PDACN) for energy‐efficient uranium extraction. The phenanthroline provides specific binding sites for selective enrichment of U(VI) at active sites (*K*_d_ = 2.79 × 10^5^ mL g^−1^ in multi‐ion solution). The Fe(II) sites have strong trap‐redox activity for U(VI) and act as dynamic electron donors to rapidly mediate electrocatalytic U(VI) extraction through the redox reaction of Fe(0/II)/Fe(III). Moreover, the Fe‐porphyrin blocks support sustained electron donation for U(VI) electrocatalysis by pre‐storing electrons. These features enable selective uranium capture and a high electroextraction capacity of 24 646.3 mg g^−1^ from simulated nuclear wastewater in 280 h at a low voltage of −1.5 V. An ultra‐high Faraday efficiency of 90.1% is achieved, and the energy cost is 3.22 × 10^−2^ $ kg^−1^ U, significantly lower than the previously reported materials. This work provides a highly efficient strategy for uranium extraction from water.

## Graphical abstract

Precise Electrocatalysis based on Ferrum (Fe) porphyrin‐phenanthroline conjugated network (Fe@PDACN) achieves energy‐efficient extraction of uranium (U). Phenanthroline enables highly selective U(VI) enrichment. Fe(II) site has strong trap‐redox activity for U(VI) and rapidly mediate electrocatalytic U(VI) extraction through Fe(0/II)/Fe(III) redox cycles. Fe‐porphyrin supports sustained electron donation for U(VI) electrocatalysis by pre‐storing electrons.

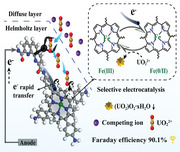

## 1 Introduction

Nuclear energy is one of the best ways to combat the global energy crisis due to its high energy density and low greenhouse gas emissions. Uranium (U) is the main raw material for fueling nuclear plants, and ensuring an adequate supply of uranium is crucial for the sustainable development of nuclear energy.^[^ ^1^ ^]^ Seawater represents a natural reservoir of uranium, boasting uranium resources ≈1000 times higher than those found on land.^[^ ^2^ ^]^ Additionally, the substantial quantity of radioactive effluents generated annually from nuclear plants and uranium mining activities also contains significant amounts of uranium. Furthermore, uranium‐containing wastewater, once leaked, are harmful to human health and the ecological environment.^[^ ^3^ ^]^ Therefore, effective extraction of uranium from aqueous solution is essential to environmental protection and the sustainable development of nuclear energy.

Multiple technologies, including adsorption,^[^ ^4^ ^]^ membrane separation,^[^ ^5^ ^]^ photocatalysis,^[^ ^6^ ^]^ have been developed for uranium extraction from seawater or radioactive effluents. However, the low uranium concentration, complex ion competition, and high salt content in these environments result in challenges of low extraction capacity, poor selectivity, and long extraction duration in uranium extraction.^[^ ^3^, ^7^ ^]^ Recently, employing electric drive and electrodeposition has emerged as a promising strategy to enhance U(VI) extraction capacity and adsorption rates.^[^ ^3^, ^7^, ^8^ ^]^ For instance, Cui and co‐workers devised a half‐wave rectified alternating current electrochemical technique for U(VI) extraction based on amidoxime‐functionalized carbon electrodes.^[^ ^3^ ^]^ Compared to physiochemical adsorption, the technique enhanced U(VI) extraction capacity and adsorption kinetics by nine and four times, respectively, at a pulse voltage of ‐5 V/0 V. As the core material of electrochemical technology, the design of high‐performance electrode materials is receiving increasing attention.^[^ ^7^, ^8^, ^9^ ^]^ Recently, Wang et al. reported a nano‐reduced iron‐based electrode materials,^[^ ^7^ ^]^ and employed the electrochemical mediated Fe(II)/Fe(III) redox reactions to achieve a uranium extraction capacity of 456.37 mg g^−1^ in seawater spiked with 20 mg L^−1^ uranium at −0.1 V. Our group have also developed a phenanthroline‐based supramolecular organic framework (MPSOF) as a cathode material,^[^ ^10^ ^]^ achieving selective and continuous electrochemical uranium extraction at −3.5 V. However, poor selectivity and low energy efficiency caused by the complex real environment and the use of high negative voltages have yet to be overcome synchronously through electrode design, ultimately diminishing extraction performance and economic benefits.

**Figure 1:**
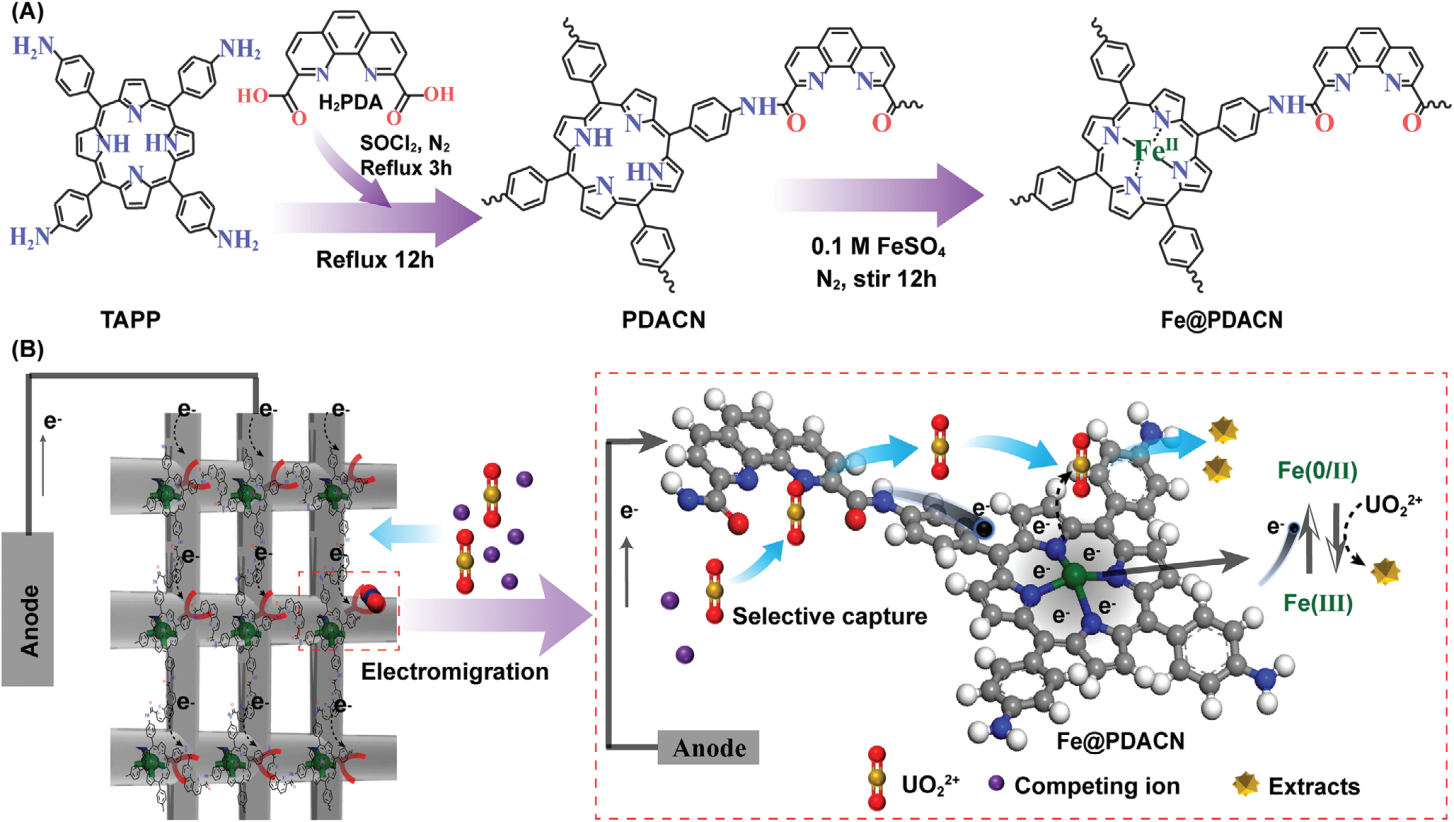
A) Synthetic route of Fe@PDACN. B) Precise electrocatalysis strategy on Fe@PDACN. The red box illustrates the precise electrocatalysis strategy, including selective capture of U(VI) and the Fe(II) sites act as a dynamic electron donor to rapidly mediate electrocatalytic U(VI) extraction through Fe(0/II) and Fe(III) redox reactions, as well as pre‐storage of electrons on Fe‐porphyrin blocks.

To address these challenges, we develop a precise electrocatalysis strategy using a Fe(II) porphyrin‐phenanthroline conjugated network (Fe@PDACN) for energy‐efficient electrocatalytic extraction of U(VI). Specifically, Fe@PDACN was prepared as an electrode material through amidation polymerization of 5,10,15,20‐tetrakis(4‐aminophenyl)‐21H,23H‐porphine (TAPP) with 1,10‐phenanthroline‐2,9‐dicarboxylic acid (H_2_PDA) and subsequent coordination with Fe(II) (**Figure** **1**A). The PDA blocks can provide specific binding sites for U(VI),^[^ ^11^ ^]^ which significantly enhances the selective enrichment of U(VI) at the active site. The Fe(II) sites have considerable trapping redox activity for uranyl ions and can act as a dynamic electron donor to rapidly mediate electrocatalytic U(VI) extraction through Fe(0/II) and Fe(III) redox reactions. Additionally, Fe‐porphyrin blocks with organometallic conjugated network structures enable pre‐storage of electrons, which assists in precise and sustained electron donation for U(VI) electrocatalysis. This electrocatalysis strategy combining uranium selective capture and high trapping electrocatalysis, as well as electron pre‐storage is expected to achieve the highly efficient U(VI) extraction at low voltages (Figure 1B). The morphology and structure of the materials were systematically characterized. The physicochemical adsorption properties and electrochemical extraction performance for uranium on Fe@PDACN were investigated. The mechanism of uranium extraction was also investigated by theoretical calculations.

## 2 Results and Discussion

### 2.1 Characterization of Fe@PDACN

**Figure 2:**
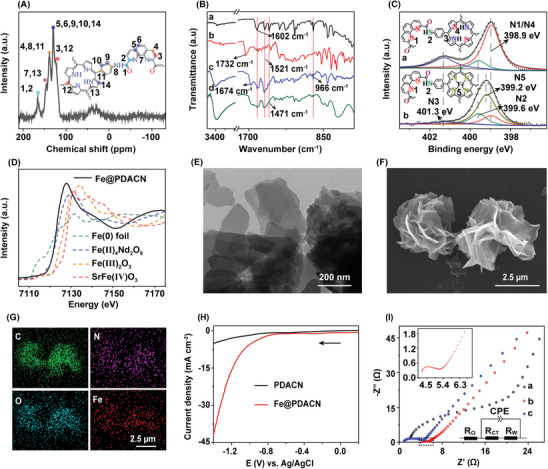
A) Solid‐state ^13^C NMR spectrum of PDACN. B) FTIR spectra of TAPP, H_2_PDA, PDACN and Fe@PDACN (a: TAPP, b: H_2_PDA, c: PDACN, d: Fe@PDACN). C) N 1s XPS spectra for PDACN and Fe@PDACN (a: PDACN, b: Fe@PDACN; The inset shows the local structural diagrams of PDACN and Fe@PDACN with four N atoms in the TAPP cavity involved in Fe coordination). D) *k*‐edge XANES spectra for Fe@PDACN, and reference substances Fe foil (0), Fe_4_Nd_2_O_9_ (divalent), Fe_2_O_3_ (trivalent) and SrFeO_3_ (tetravalent). E) TEM image of Fe@PDACN. F) SEM image of Fe@PDACN, and G) corresponding EDS maps for the elements C, N, O, and Fe. H) Linear sweep voltammetry curves of PDACN and Fe@PDACN. I) Electrochemical impedance spectroscopy of PDACN, Fe@PDACN and blank conductive carbon cloth (a: blank conductive carbon cloth, b: Fe@PDACN, c: PDACN).

The chemical structures of the materials were characterized by solid‐state ^13^C NMR, Fourier transform infrared (FTIR) spectroscopy, X‐ray photoelectron spectroscopy (XPS), and energy−dispersive spectroscopy (EDS). The solid‐state ^13^C NMR spectrum of PDACN shows that a small peak corresponding to the amide carbon atom and the C‐2 of phenanthroline fraction was found at 166 ppm (Nos. 1, 2 in the inset) (**Figure** **2**A).^[^ ^12^ ^]^ Several peaks in the δ range of 120–160 ppm are attributed to the aromatic carbon atoms of the phenanthroline and porphyrin fractions (Nos. 3–14 in the inset).^[^ ^13^ ^]^ As shown in the FTIR spectra (Figure 2B), the characteristic band at 1732 cm^−1^ is attributed to the C═O of the carboxyl group on H_2_PDA. It shifted to 1674 cm^−1^ after the reaction, corresponding to the C═O stretching vibration on the amide group, indicating the successful polymerization of TAPP and H_2_PDA.^[^ ^14^ ^]^ The bands at 1602, 1521, 1471 cm^−1^ are identified as the C═C, C═N stretching vibrations on the porphyrin ring.^[^ ^14^, ^15^ ^]^ In addition, the band at 966 cm^−1^ corresponds to the in‐plane bending vibration of N─H,^[^ ^13^ ^]^ which disappears after the coordination between porphyrin with Fe. The XPS spectra of Fe@PDACN show that Fe 2p peaks can be found at 711.5 and 725.3 eV (Figure S1, Supporting Information), and the Fe content of the material was determined to be 5.4 wt.% and 5.2 wt.% by EDS and ICP‐MS, respectively. All the results indicate the successful loading of Fe.

Subsequently, the coordination environment and valence states of Fe was analyzed. Figure 2C shows that the N1s spectrum of PDACN can be deconvolved into three peaks at 398.9, 399.6, and 401.3 eV, corresponding to pyridine‐like N (N1/N4), sec‐amide N (N2), and sec‐amine (N3), respectively.^[^ ^11^, ^16^ ^]^ The N3 and N4 peaks were greatly reduced and replaced by a new Fe─N peak (399.2 eV) after Fe loading, suggesting that the four N atoms in the TAPP are equivalently involved in the coordination of Fe.^[^ ^17^ ^]^ The Fourier transform (FT) Extended X‐ray absorption fine structure (EXAFS) spectra of Fe@PDACN and their fitting results show that the Fe─N coordination number is 4.25, further confirming that the Fe‐porphyrin structures in Fe@PDACN, which has a Fe─N length of 2.06(2) Å (Figure S3 and Table S1, Supporting Information). The absorption edge X‐ray absorption near edge structure (XANES) spectra show that the energy position of Fe@PDACN was closer to Fe_4_Nd_2_O_9_ (divalent) compared to other reference substances (Figure 2D), indicating that Fe in Fe@PDACN is mostly divalent. Considering that Fe(II) and Fe(III) can be electrochemically reduced at the cathode,^[^ ^7^ ^]^ both Fe(II) and Fe(III) species can be used as active catalytic sites for electrocatalytic processes.

The morphology and microstructure of the materials were mainly characterized by scanning electron microscopy (SEM), transmission electron microscopy (TEM), atomic force microscopy (AFM) and powder X‐ray diffraction (PXRD). SEM and TEM images of PDACN show a lamellar assembly morphology, and EDS maps show that the elements C, N, and O were all uniformly dispersed in PDACN (Figure S4, Supporting Information). After coordination with Fe(II), Fe@PDACN shows a petal‐like lamellar assembly morphology (Figure 2E,F), and EDS mapping (Figure 2G) shows that the elements C, N, O, and Fe elements were uniformly distributed. AFM images further confirm the lamellar morphology of Fe@PDACN, which has a monolithic thickness of ≈1.7 nm (Figure S5, Supporting Information). PXRD patterns show low crystallinity of PDACN and Fe@PDACN (Figure S6, Supporting Information). The specific surface areas of PDACN and Fe@PDACN were 8.0 and 10.7 m^2^ g^−1^, respectively (Figure S7 and Table S2, Supporting Information). The low specific surface areas are probably due to the low ordered structure of PDACN and Fe@PDACN. The lamellar assembly morphology of PDACN and Fe@PDACN contributes to enhance the exposure of active sites on the interface of the materials,^[^ ^18^ ^]^ thus promoting the interaction between the uranium and material interfaces, which is conducive to achieving rapid uranium adsorption and high active site utilization.

Considering that acidic conditions are a common environment for radioactive wastewater, Fe@PDACN was immersed with 1 m HNO_3_ for 24 h to assess the acid stability of the material. SEM image and FTIR spectra show that Fe@PDACN maintains its original petal‐like lamellar assembly morphology and chemical structure (Figure S8, Supporting Information). The Fe loading measured by ICP‐MS was maintained at 5.1 wt.%. These results indicate that Fe@PDACN has good acid stability, which is attributed to the highly chemically stability of large conjugated backbone and macrocyclic coordination iron porphyrin.^[^ ^19^ ^]^

The electrocatalytic activity of Fe@PDACN was analyzed by linear sweep voltammetry (LSV) and electrochemical impedance spectroscopy (EIS) in 100 mg L^−1^ of UO_2_^2+^ solution. As shown in Figure 2H, the onset potential of Fe@PDACN (−0.66 V vs Ag/AgCl) was more positive than that of the PDACN (−0.84 V vs Ag/AgCl), and the current density of Fe@PDACN was higher than that of PDACN. These results indicate that the introduction of Fe─N coordination endows Fe@PDACN with a higher electrocatalytic reduction activity toward uranyl ions. This is due to the high electron conductivity of metal coordination bonds and the conjugated structure (including p–π and π–π conjugation) of the PDACN network. To confirm this point, the interfacial reactivity of Fe@PDACN‐loaded electrode, PDACN functionalized electrode and conductive carbon cloth was analyzed by EIS. Figure 2I shows that the charge transfer resistance (*R*_CT_) of the PDACN functionalized electrode (4.2 Ω) is much lower than that of the blank conductive carbon cloth (14.1 Ω). This can be attributed to the delocalized electrons within the conjugated network, which facilitates the electron transport process. After the coordination of PDACN with Fe, the *R*_CT_ of the Fe@PDACN‐loaded electrode further decreases to 1.3 Ω, only one third of that of the PDACN functionalized electrode. This decrease in *R*_CT_ suggests that the Fe‐porphyrin blocks on the Fe@PDACN promote interfacial electron transfer, thereby facilitating the electroreduction of U(VI).

### 2.2 Physicochemical Adsorption of Uranium

**Figure 3:**
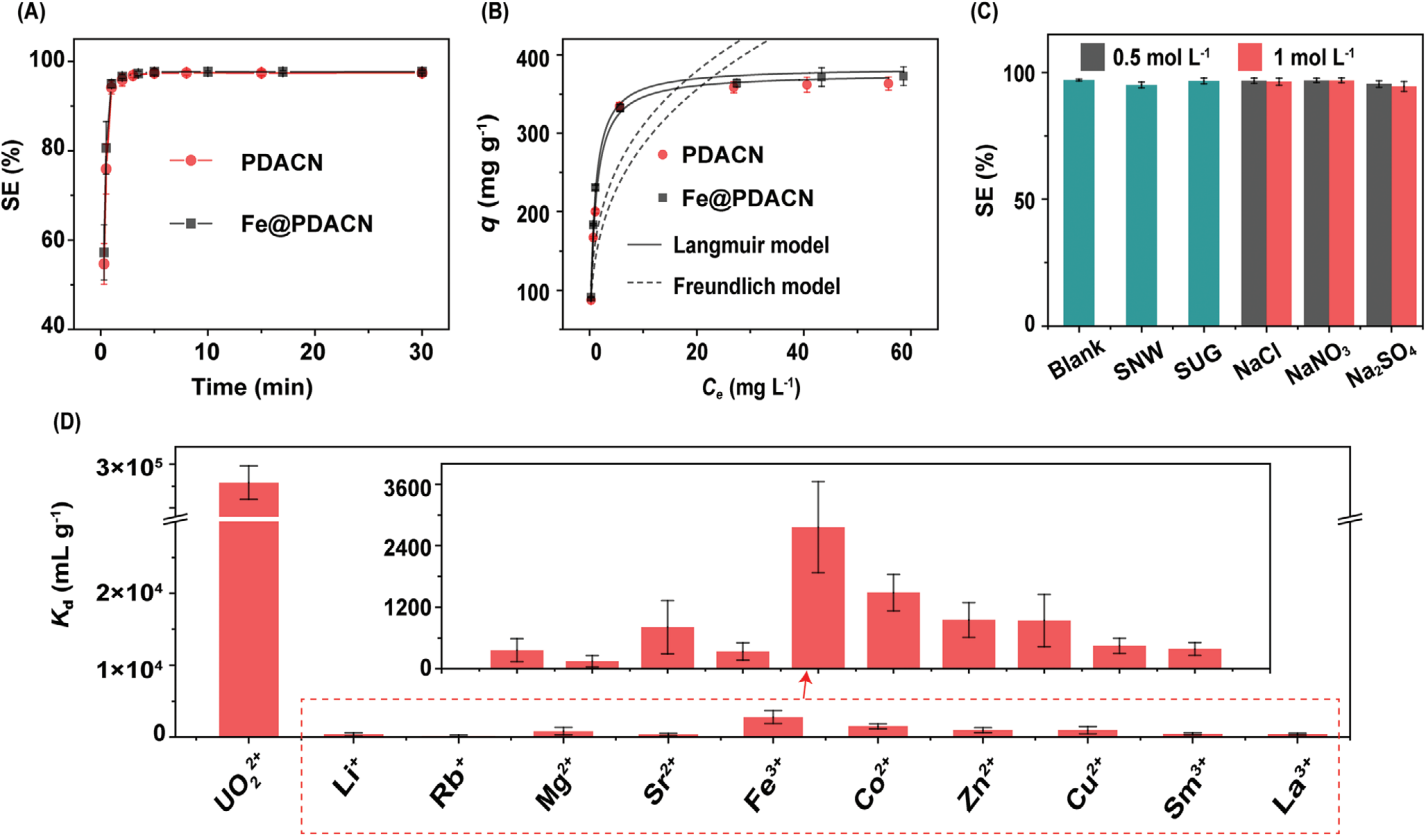
A) Sorption kinetics of PDACN and Fe@PDACN for U(VI). Conditions: solid–liquid ratio = 0.1 g L^−1^, initial *C*_U(VI)_ = 11.9 mg L^−1^. B) Sorption isotherms of PDACN and Fe@PDACN for U(VI). Conditions: solid–liquid ratio = 0.1 g L^−1^. C) Adsorption efficiency for U(VI) onto Fe@PDACN in different solutions. Conditions: solid–liquid ratio = 1 g L^−1^, initial *C*_U(VI)_ = 50 mg L^−1^ in blank and inorganic salt solutions (SNW: simulated nuclear wastewater, *C*_U(VI)_ = 50 mg L^−1^, Table S6, Supporting Information; SUG: simulated uranium‐containing groundwater, *C*_U(VI)_ = 1.38 mg L^−1^, Table S7, Supporting Information). D) Distribution ratio (*K*_d_) of Fe@PDACN for UO_2_^2+^ and other metal ions. Conditions: solid–liquid ratio = 0.1 g L^−1^, initial *C*_U(VI)_ = *C*_metal ion_ = 1 × 10^−4^ mol L^−1^.

The physicochemical adsorption performance of Fe@PDACN for U(VI) was investigated to demonstrate its potential for selective enrichment of U(VI). PDACN and Fe@PDACN show high adsorption efficiencies of over 90% across a pH range of 1.0–8.0 (Figure S9A, Supporting Information). At the same time, we notice that both PDACN and Fe@PDACN exhibit the same surface charge characteristics as the major uranium species in the pH ranges of 1.0–4.0 and 8.0 (Figure S9B,C, Supporting Information), which typically leads to electrostatic repulsion between the adsorbent and the adsorbate. Therefore, electrostatic interactions do not play a dominant role in uranium adsorption. This pH‐independent adsorption behavior of the materials is mainly attributed to the abundant PDA ligands in the materials, which have a high affinity for uranium.^[^ ^11^, ^20^ ^]^ **Figure** **3**A shows that Fe@PDACN and PDACN have similar adsorption kinetics, removing more than 80% of the U(VI) within the initial 30 s and reaching equilibrium within 5 min. The adsorption behaviors were all well described by pseudo‐second‐order kinetic models (Figure S10 and Table S3, Supporting Information), suggesting that chemical sorption was the rate‐limiting step. The adsorption isotherms of PDACN and Fe@PDACN were well fitted by the Langmuir isotherm model, indicating monolayer adsorption with a uniform distribution of adsorption sites (Figure 3B; Table S4, Supporting Information).^[^ ^21^ ^]^ The maximum adsorption capacities of PDACN and Fe@PDACN were 376.4 and 384.0 mg g^−1^, respectively, which are considerable values compared to the previously reported sorbents (Table S5, Supporting Information). Both PDACN and Fe@PDACN retained their lamellar morphology after U(VI) adsorption (Figures S11 and S12, Supporting Information).

The selectivity of Fe@PDACN was investigated in high‐concentration saline solutions and various simulated uranium‐containing wastewaters, including simulated nuclear wastewater (SNW, Table S6, Supporting Information)^[^ ^1^, ^22^ ^]^ and simulated uranium‐containing groundwater (SUG, Table S7, Supporting Information).^[^ ^23^ ^]^ As shown in Figure 3C, Fe@PDACN remained a high adsorption efficiency for U(VI) in high‐concentration saline solutions and various simulated uranium‐containing wastewaters, compared to that observed in a single uranium solution. Furthermore, Figure 3D shows that Fe@PDACN has a fairly high U(VI) distribution ratio (*K*_d,U_) of 2.79 × 10^5^ mL g^−1^ in the multi‐ion solution, significantly surpassing other reported materials, which highlights its superior affinity for uranium.^[^ ^9^, ^24^ ^]^ All the selectivity coefficients *β* (*K*_d_, _U_/*K*_d_, _ion_) (Table S9, Supporting Information) exceed 100, indicating an excellent selectivity of Fe@PDACN for U(VI). This high selectivity is expected to address the drawback of the electric field's indiscriminate attraction to all ions, thereby enhancing the selectivity of electroextraction for uranium.

### 2.3 Electrochemical Extraction

Fe@PDACN was loaded onto hydrophilic conductive carbon cloth by drop‐coating approach using Nafion solution as a binder to obtain Fe@PDACN‐loaded electrode (Figure S13, Supporting Information). The adsorbent loading per electrode was ≈2.0 mg. The Fe@PDACN‐loaded electrode shows a good hydrophilicity with a low water contact angle of 26° (Figure S13D, Supporting Information). The electrochemical U(VI) extraction on Fe@PDACN was optimized via applied voltage and initial U(VI) concentration. PDACN loaded conductive carbon cloth was also prepared as a reference. The feasibility of Fe@PDACN‐based electrochemical uranium extraction in practical applications was investigated in simulated uranium‐containing wastewater and natural seawater.

#### 2.3.1 Effect of Voltage

**Figure 4:**
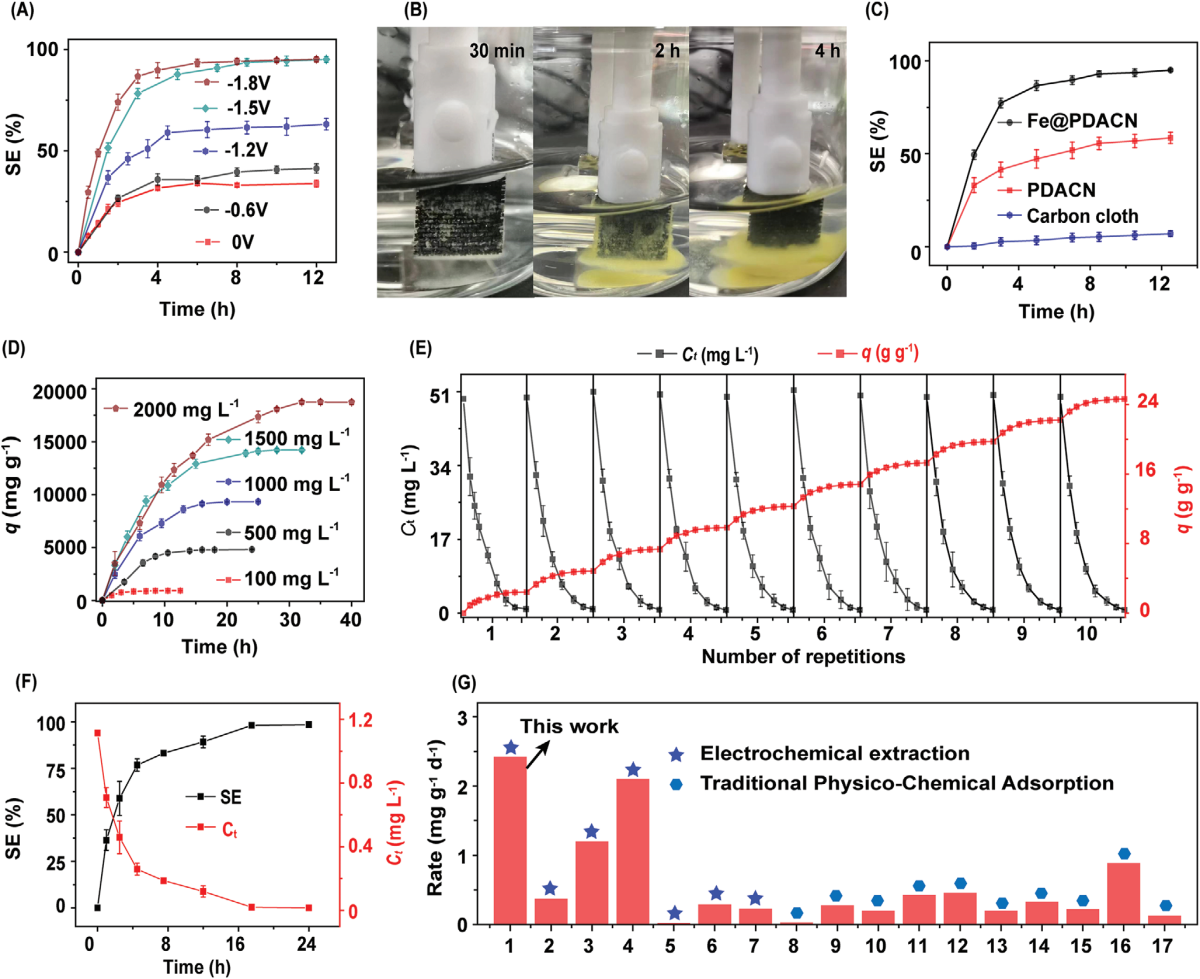
A) Performance of electrochemical U(VI) extraction at different constant voltages and B) corresponding photographs during electrochemical U(VI) extraction at −1.5 V. Conditions: solid–liquid ratio = 0.1 g L^−1^, initial *C*_U(VI)_ = 100 mg L^−1^, *C*_NaCl_ = 1 mol L^−1^. C) Electrochemical U(VI) extraction performance of Fe@PDACN, PDACN and blank carbon cloth. Conditions: solid–liquid ratio = 0.1 g L^−1^, initial *C*_U(VI)_ = 100 mg L^−1^, voltage = −1.5 V, *C*_NaCl_ = 1 mol L^−1^. D) Electrochemical extraction amount of U(VI) at different initial U(VI) concentrations. Conditions: solid–liquid ratio = 0.1 g L^−1^, voltage = −1.5 V, *C*_NaCl_ = 1 mol L^−1^. E) Continuous electrochemical U(VI) extraction in SNW without elution. Conditions: solid–liquid ratio = 0.02 g L^−1^, voltage = −1.5 V, initial *C*_U(VI)_ = 50 mg L^−1^. F) Uranium extraction from spiked seawater with initial uranium concentrations of ≈1 mg L^−1^. Conditions: solid–liquid ratio = 0.01 g L^−1^, using a 1.5 V/0 V square wave with a frequency of 1 Hz. G) Comparison of the uranium extraction rate from natural seawater of Fe@PDACN with other materials. From left to right were: this work, BSA@CFF,^[^ ^26^ ^]^ Fe‐N_x_‐C‐R,^[^ ^8^ ^]^ B:Cu‐PO_4_ nanoparticle,^[^ ^9^ ^]^ TETA‐PAO/GF,^[^ ^27^ ^]^ MIGPAF‐13,^[^ ^9^ ^]^ PPA@MISS‐PAF‐1,^[^ ^28^ ^]^ PAO‐G‐A,^[^ ^29^ ^]^ PAO‐PNM,^[^ ^4^ ^]^ PAO‐Co,^[^ ^30^ ^]^ VA‐PG,^[^ ^31^ ^]^ MUU,^[^ ^32^ ^]^ AO‐OpNpNc fibers,^[^ ^33^ ^]^ Zn^2+^–PAO hydrogel,^[^ ^34^ ^]^ NDA‐TN‐AO,^[^ ^6^ ^]^ UiO‐66‐AO,^[^ ^35^ ^]^ H‐ABP fiber.^[^ ^4^ ^]^

The effect of different voltages (0, −0.6, −1.2, −1.5, −1.8 V) on the performance of electrochemical U(VI) extraction was initially investigated. As shown in **Figure** **4**A, the adsorption efficiency of the electrochemical U(VI) extraction increases significantly with decreasing voltage. Notably, a large amount of yellow precipitate formed near the Fe@PDACN‐loaded electrode when the voltage was ≤−1.5 V (Figure 4B), illustrating the occurrence of the electroredox reaction of U(VI). The electrochemical U(VI) extraction performance of Fe@PDACN, PDACN and blank carbon cloth was investigated at −1.5 V (Figure 4C). After 12.5 h of electrochemical U(VI) extraction, the extraction efficiency for U(VI) on blank carbon cloth was less than 10% (68.6 mg g^−1^), whereas the extraction efficiencies of PDACN and Fe@PDACN were 58.6% (574.4 mg g^−1^) and 95.0% (931.2 mg g^−1^), respectively. These results verified the enhancing effect of the conjugated network and Fe(II) catalytic sites on the electrochemical U(VI) extraction.

#### 2.3.2 Effects of Initial Uranium Concentration

The extraction efficiency and amount of the Fe@PDACN‐based electrochemical U(VI) extraction were determined with various initial U(VI) concentrations. As illustrated in Figure S14 (Supporting Information) and Figure 4D, as the initial U(VI) concentration increased from 100 to 2000 mg L^−1^, the equilibrium extraction efficiency remained constant (≈97.7%), while the extraction amount surged from 923.6 to 18 766.7 mg g^−1^. This electroextraction amount far exceeds the maximum physicochemical adsorption capacity of Fe@PDACN (384.0 mg g^−1^), reaching ≈49 times. The constant extraction efficiency in varied uranium concentration indicates that the Fe@PDACN‐based electrochemical U(VI) extraction has an infinite extraction capacity for U(VI), which is attributed to the self‐detachment of the U(VI) precipitate generated by the electroredox reaction from Fe@PDACN and regenerated the active sites (Video S1, Supporting Information).

#### 2.3.3 Continuous Electrochemical Extraction and Energy Consumption Analysis

To confirm the refer of infinite U(VI) extraction capability by Fe@PDACN‐based electrochemical U(VI) extraction and the reusability of the Fe@PDACN‐loaded electrode, ten consecutive (280 h) elution‐free electrochemical U(VI) extractions were performed in SNW (initial *C*_U(VI)_ = 50 mg L^−1^). Each extraction was carried out until the U(VI) concentration was <1 mg L^−1^, i.e., more than 98% of U(VI) was recovered (Figure S15, Supporting Information). Figure 4E demonstrates that Fe@PDACN can effectively electrochemically extract U(VI) from SNW, and more significantly, there is no decline in the performance of electrochemical U(VI) extraction for ten consecutive times. After 280 h of the electrochemical U(VI) extraction, the U(VI) extraction amount increased to 24 646.3 mg g^−1^. Compared to other high performance electrode materials, Fe@PDACN is able to achieve much higher electrochemical U(VI) extraction performance at low voltage (−1.5 V) (Table S10, Supporting Information). Moreover, SEM image and FTIR spectra show no significant changes in the morphology and chemical structure of Fe@PDACN‐loaded electrodes after repeated use (Figure S16A,B, Supporting Information), as compared to the fresh Fe@PDACN‐loaded electrodes (Figure S13C, Supporting Information). Furthermore, EDS analysis (Figure S16C, Supporting Information) confirmed that the Fe content remained stable after ten consecutive electrochemical extractions, and Fe 2p XPS spectra of the used electrodes indicated that Fe(II) remained the predominant form of Fe sites (Figure S16D, Supporting Information), consistent with the fresh Fe@PDACN (Figure 2D). This high electrocatalytic stability can be attributed to the good chemical stability of large conjugated backbone and high electron‐mediated activity of iron porphyrin.^[^ ^19^, ^25^ ^]^ These results reveal that Fe@PDACN has infinite electrochemical U(VI) extraction capacity and good electrocatalytic stability.

The energy cost and Faraday efficiency of Fe@PDACN‐based electrochemical U(VI) extraction were also evaluated (Table S11, Supporting Information). The current was monitored by chronoamperometry every three hours for 1000 s to ensure that the current was stable (Figure S17, Supporting Information). The current‐time curves were integrated to calculate an average current of 8.8 × 10^−5^ A. The price of electricity used was ≈8.6 ¢/kWh (Shanghai commercial average electricity prices in 2023 from State Grid Corporation of China). The energy cost of Fe@PDACN‐based electrochemical U(VI) extraction is 3.22 × 10^−2^ $ kg^−1^ U, which is much lower than previously reported materials (Table S12, Supporting Information). Furthermore, the Faraday efficiency of Fe@PDACN‐based electrochemical U(VI) extraction reaches an impressive 90.1%, indicating exceptionally high efficiency in electricity utilization. The infinite extraction and ultra‐high Faraday efficiency feature of Fe@PDACN‐based electrochemical U(VI) extraction present substantial cost savings and environmental benefits, rendering it a promising technology for engineering U(VI) extraction applications.

#### 2.3.4 Electrochemical Extraction from Simulated Groundwater and Natural Seawater

In view of the excellent electroextraction performance of Fe@PDACN described above, we further tested the uranium extraction performance in simulated uranium‐containing groundwater (Table S7, Supporting Information), uranium‐spiked seawater (≈1 mg L^−1^) and natural seawater (≈3.3 µg L^−1^). The amount of Fe@PDACN‐based electrochemical U(VI) extraction in simulated groundwater (*C*_U(VI)_ = 1.38 mg L^−1^) reached 824.4 mg g^−1^ within 205 h, which is 2.2 times the physicochemical adsorption capacity of Fe@PDACN (Figure S18, Supporting Information).

As shown in Figure 4F, Fe@PDACN removed 89.3% and 98.5% of uranium from seawater samples spiked with 1 mg L^−1^ uranium within 12 and 24 h, respectively. The extraction amount was 109 mg g^−1^ within 24 h. The extraction amount was 109 mg g^−1^ within 24 h. Subsequently, the electrochemical uranium extraction performance of Fe@PDACN was evaluated in natural seawater, where it successfully extracted 9.6 µg of uranium from 4 L of natural seawater, yielding a calculated extraction rate of 2.4 mg g^−1^ d^−1^. Comparative analysis with state‐of‐the‐art materials in recently reported literature^[^ ^4^, ^6^, ^8^, ^9^, ^26^, ^27^, ^28^, ^29^, ^30^, ^31^, ^32^, ^33^, ^34^, ^35^ ^]^ indicates that Fe@PDACN stands out as one of the best performing adsorbents available for uranium extraction from natural seawater (Figure 4G and Table S13, Supporting Information), demonstrating exceptional extraction performance. As a result, Fe@PDACN exhibits significant potential for the electrochemical extraction of from seawater and uranium‐containing groundwater.

### 2.4 Mechanism Studies

#### 2.4.1 Precise Electrocatalysis

**Figure 5:**
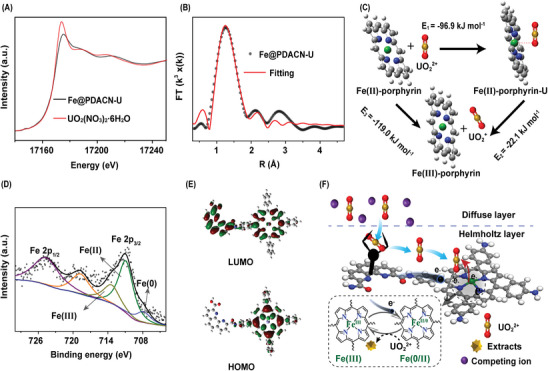
A) U L_3_‐edge XANES spectra for Fe@PDACN‐U (Fe@PDACN after physicochemical adsorption), and UO_2_(NO_3_)_2_·6H_2_O. B) U L_3_‐edge EXAFS R‐space and corresponding fitting curves for Fe@PDACN‐U. C) Free energy of the Fe‐porphyrin block adsorption and reduction of U(VI). D) Fe 2p XPS spectra of Fe@PDACN‐loaded electrode after the electrochemical uranium extraction. E) HOMO and LUMO distribution of Fe@PDACN core fragments. F) Schematic illustrating the precise electrocatalysis mechanism.

In order to fully understand the internal factors underlying the infinite extraction and ultra‐high Faraday efficiency of Fe@PDACN‐based electrochemical U(VI) extraction, mechanistic studies were carried out. The near‐edge feature of Fe@PDACN after physicochemical adsorption was consistent with that of uranyl nitrate hexahydrate, as shown in their XANES spectra, indicating that the adsorbed uranium is UO_2_^2+^ (**Figure** **5**A). The EXAFS spectra (Figure 5B) and corresponding fitting results (Figure S19A and Table S14, Supporting Information) reveal that each uranyl ion is coordinated to two oxygen atoms from two H_2_O molecules as well as two oxygen and two nitrogen atoms from the PDA block (Figure S19B, Supporting Information).^[^ ^36^ ^]^ This chelate structure has much higher stability compared to chelate structures of PDA with other metal ions,^[^ ^20^, ^37^ ^]^ which is the underlying reason for the high selectivity of Fe@PDACN toward U(VI). Thus, according to Langmuir adsorption theory,^[^ ^21^ ^]^ the U(VI) coming from the electric field drive will be further selectively enriched at the active sites during the dynamic process of adsorption–desorption.

DFT calculations were performed for the reaction thermodynamics associated with the spontaneous reduction of U(VI) by the Fe(II)‐porphyrin block. Taking into account of the possible spin states, the calculated single point energies for the optimized structures are listed in Table S15 (Supporting Information). These single point energies were used to calculate the adsorption energy (*E*_1_) and the reductive desorption energy (*E*_2_) of U(VI) at the Fe‐porphyrin blocks, which were −96.9 and −22.1 kJ mol^−1^, respectively (Figure 5C). The negative free energy indicates that the Fe‐porphyrin block has the inherent ability to spontaneously adsorb and reduce enriched U(VI). Surface‐mediated reduction by Fe(II) has been generally regarded as an efficient pathway for the immobilization of some radionuclides.^[^ ^38^ ^]^ Figure S20 (Supporting Information) shows that U(IV) in Fe@PDACN‐U accounted for 7.3 wt.% of the total extracted uranium, and Fe(II) in Fe@PDACN accounted for 3.4 wt.% of the total mass. The low percentage content of Fe(II) in Fe@PDACN resulted in its limited capacity to reduce U(VI). This supports the fact that Fe@PDACN (384.0 mg g^−1^) and PDACN (376.4 mg g^−1^) have similar adsorption capacities in physicochemical adsorption (Figure 3B; Table S4, Supporting Information). The limited reduction capacity in physicochemical adsorption contrasts with the infinite extraction capacity in electrocatalytic extraction. Therefore, the abundant Fe(0/II) sites and PDA blocks construct a selective trap‐electrocatalysis platform, which provides the basis for energy‐efficient electrocatalytic extraction of uranium at low voltage.

The valence state of Fe in the materials after electrochemical uranium extraction was analyzed by XPS to assess the regeneration of catalytic site. The fine spectra of Fe 2p reveal three valence states of Fe on the Fe@PDACN after electrochemical uranium extraction: Fe (0), Fe (II), and Fe (III), located at 707.9, 711.1, and 713.4 eV, respectively (Figure 5D). This observation suggests that Fe(0/II) can be regenerated and used for the electrocatalytic reduction of U(VI) during the electrochemical uranium extraction process. Furthermore, Density Functional Theory (DFT) calculations show that Fe@PDACN has a higher HOMO level and a lower HOMO‐LUMO gap (−5.04, 2.53 eV) compared to TAPP (−6.33, 4.01 eV) and PDACN (−5.08, 2.65 eV), indicating a higher electron donating ability and conductivity of Fe@PDACN (Figure S21, Supporting Information).^[^ ^39^ ^]^ This result aligns with the observations from electrochemical impedance spectroscopy (Figure 2I). As depicted in Figure 5E, the HOMO of Fe@PDACN is predominantly localized around the Fe‐porphyrin rings, highlighting their crucial role in electronstorage and donation.^[^ ^40^ ^]^ Therefore, Fe‐porphyrin with an organometallic conjugated network structure enables the pre‐storage of electrons, while the Fe(II) site acts as a dynamic electron donor to rapidly mediate electrocatalytic U(VI) extraction via redox reactions of Fe(0/II) and Fe(III).

Thus, the precise electrocatalysis strategy as shown in Figure 5F: First, the PDA blocks enrich U(VI) highly selectively into the Helmholtz layer of the electrical double layer when the electric field indiscriminately enriches all ions, reducing the intervention of interfering ions. Second, The Fe(II) sites have strong trap‐redox activity for U(VI) and act as a dynamic electron donor to rapidly mediate the electrocatalytic extraction of U(VI) through redox reactions of Fe(0/II) and Fe(III). In addition, Fe‐porphyrin blocks with organometallic conjugated network structures enable pre‐storage of electrons, which assists in precise and sustained electron donation for U(VI) electrocatalysis. This precise electrocatalysis strategy exploits uranium specific capture, high trap‐electrocatalysis and electron pre‐storage underpins the high performance of Fe@PDACN‐based electrochemical U(VI) extraction.

#### 2.4.2 Uranium Electrodeposition

**Figure 6:**
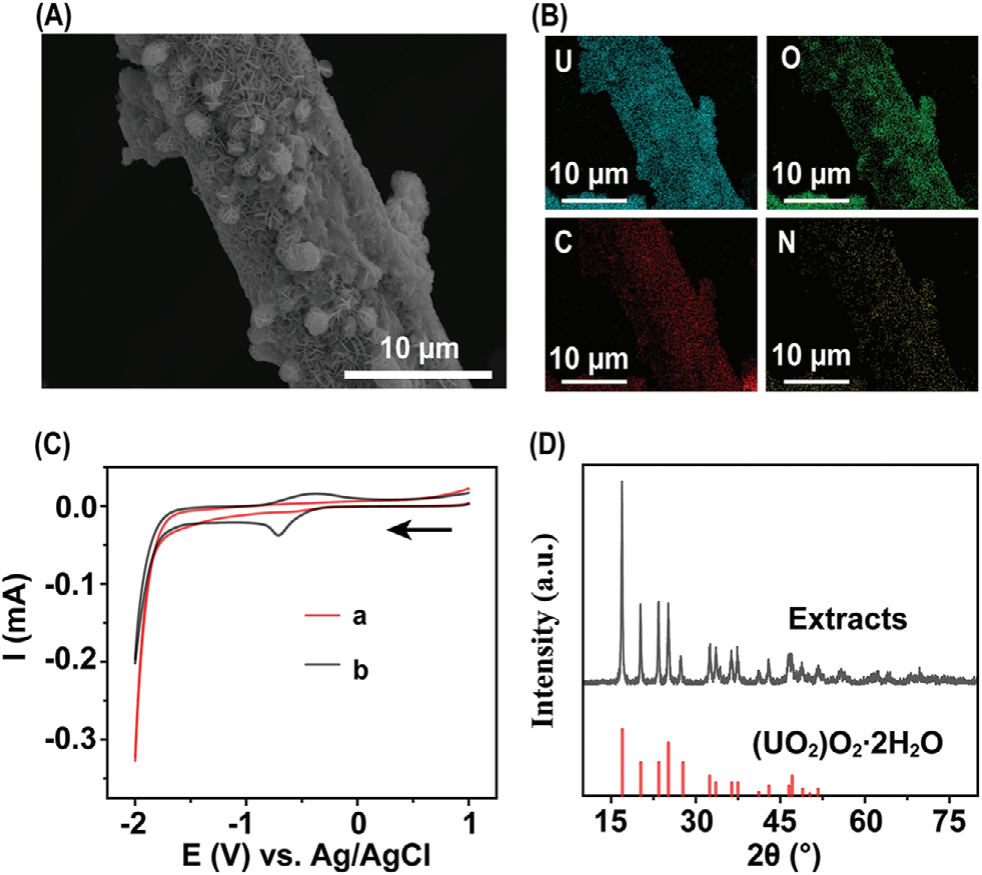
A) SEM images of electrodeposits grown on Fe@PDACN‐loaded electrode, and B) corresponding EDS maps for the elements U, O, C, and N. C) Cyclic voltammograms of uranyl‐nitrate‐spiked 1 m NaCl solution compared to 1 m NaCl solution (a: 1 m NaCl, b: 1 m NaCl with U(VI) concentrations of 100 mg L^−1^). D) XRD patterns of electrochemical extracts under air and XRD peaks of reference (UO_2_)O_2_·2H_2_O (JCPDS 01‐081‐9033).

The species transformation of uranium on Fe@PDACN during electrochemical extraction were investigated vis SEM, EDS, cyclic voltammetry (CV), XRD, and Raman spectroscopy. **Figure** **6**A,B shows the uniform growth of uranium deposits on the electrode surface during the initial electrodeposition process (10 min). Figure 6C shows that in a 1 m NaCl aqueous solution spiked with 100 mg L^−1^ U(VI), a reduction peak of U(VI) to U(V) (−0.71 V vs Ag/AgCl) and an oxidation peak of U(V) to U(VI) (−0.38 V vs Ag/AgCl) were observed.^[^ ^8^ ^]^ The peak current of the oxidation peak of 1.594 × 10^−5^ A is lower in absolute value than the peak current of the reduction peak of −3.788 × 10^−5^ A, which may be due to the disproportionation reaction of some of the U(V).^[^ ^3^ ^]^ XRD analysis identified the electrodeposits as (UO_2_)O_2_·2H_2_O species (JCPDS 01‐081‐9033) (Figure 6D). This could be attributed to the redox processes induced by H_2_O_2_, generated from dissolved oxygen by the electric field.^[^ ^3^ ^]^ To verify this hypothesis, electrochemical extraction was performed in an N_2_ atmosphere. No visible electrodeposition was observed after 12 h of energization (Figure S22, Supporting Information). In contrast, yellow electrodeposits were observed in the testing solution containing H_2_O_2_ under a N_2_ atmosphere after ≈1 min (Video S2, Supporting Information), which occurred significantly faster than in the air atmosphere (≈20 min). This accelerated deposition probably results from a rapid interaction between excess H_2_O_2_ and U(V). XRD and Raman spectroscopy analyses support the participation of H_2_O_2_ in the transformation of uranium species induced by a redox reaction (Figure S23, Supporting Information), which is similar to previous literature.^[^ ^3^ ^]^

## 3 Conclusion

In summary, we developed a precise electrocatalysis strategy using a Fe(II) porphyrin‐phenanthroline conjugated network (Fe@PDACN) for energy‐efficient extraction of uranium. The incorporation of PDA blocks imparted Fe@PDACN with high U(VI) affinity (*K*_d_ = 2.79 × 10^5^ mL g^−1^), which significantly enhances the selective enrichment of U(VI) at the active site. Fe(II) sites exhibit considerable trap‐redox activity toward uranyl ions and act as dynamic electron donors to rapidly mediate electrocatalytic U(VI) extraction through Fe(0/II) and Fe(III) redox reactions. Fe‐porphyrin with organometallic conjugated network structures can pre‐store electrons, which assists in precise and sustained electron donation for U(VI) electrocatalysis. Detailed electrochemical extraction tests showed that Fe@PDACN serves as a stable and efficient electrocatalyst for uranium resource recovery with infinite extraction capacity and ultra‐high Faraday efficiency (up to 90.1%). The energy cost is 3.22 × 10^−2^ $ kg^−1^ U, which is much lower than the previously reported materials, indicating a huge potential in uranium extraction from water. Moreover, Fe@PDACN extracted 9.6 µg of uranium from 4 L of natural seawater with a considerable extraction rate of 2.4 mg g^−1^ d^−1^.

The precise electrocatalysis strategy demonstrates highly precise and infinite uranium extraction performance, achieving ultra‐high Faradaic efficiency and low energy consumption. This approach can also be extended to the efficient extraction of other metal ions or to other fields of electrocatalysis. The concept of precise electrocatalysis and the electrode construction method presented in this work shed light on the design of advanced electrocatalysts for various applications.

## 4 Experimental Section

### Synthesis of Fe@PDACN

H_2_PDA (670 mg, 2.5 mmol) was added to a three‐necked flask containing 35 mL SOCl_2_, and 3 drops of DMF were added as catalyst, then refluxed at 90 °C for 3 h under N_2_ atmosphere. The residual SOCl_2_ solvent was removed by reduced pressure distillation after cooling. The yellow solid was dissolved in CH_2_Cl_2_ (20 mL) without further purification and recorded as an acyl chloride solution. TAPP (673.8 mg, 1 mmol) was added to a three‐necked flask containing CH_2_Cl_2_ (20 mL) and Et_3_N (2 mL) and sonicated for 10 min, then the previous acyl chloride solution was added and refluxed at 70 °C overnight under N_2_ atmosphere. Following cooling and filtration, the sediments were washed three times with CH_2_Cl_2_ and deionized water, then freeze‐dried. The PDACN acquired was stored in a moisture‐proof cabinet.

The aqueous solution of 0.1 m FeSO_4_ (30 mL) was mixed with PDACN (500 mg) and bubbled with N_2_ for 30 min. The mixture was then sealed and shaken overnight. The solid was washed three times with deionized water after filtration and freeze‐dried. The Fe@PDACN acquired was stored in a moisture‐proof cabinet.

### Electrochemical Measurements

All electrochemical measurements were performed on an electrochemical workstation (CHI660E) in a three‐electrode configuration at room temperature. Fe@PDACN (or PDACN) functionalized electrodes, Ag/AgCl electrodes and platinum sheets were used as working, reference and counter electrodes, respectively. Electrochemical impedance spectroscopy (EIS) was carried out from 100 kHz to 1 Hz in a solution containing 100 mg L^−1^ U and 1 m NaCl at an open circuit potential of 0.23 V and an amplitude of 5.0 mV. Linear scanning voltammetry (LSV) was performed in a solution containing 100 mg L^−1^ U and 1 m NaCl over a scan range of 0 to −1.5 V at a scan rate of 20 mV s^−1^.

### Electrochemical Extraction of Uranium

All electrochemical uranium extraction experiments were performed in a standard two‐electrode system using a platinum sheet as the anode and a Fe@PDACN (or PDACN) functionalized electrode as the cathode. The two electrodes are spaced 1.2 cm apart and are parallel to each other face to face. The constant potential electrical signal is output through the function/arbitrary waveform generator (RIGOL, DG1022Z). The solution samples were filtered through a 0.45 µm membrane filter and diluted. The final uranium content was determined using inductively coupled plasma optical emission spectroscopy (ICP‐OES) or inductively coupled plasma mass spectrometry (ICP‐MS).

## Conflict of Interest

The authors declare no conflict of interest.

## Supporting information



Supporting Information

Supplemental Video 1

Supplemental Video 2

## Data Availability

The data that support the findings of this study are available from the corresponding author upon reasonable request.
