# Supplementary material for: Precise Electrocatalysis on Fe‐Porphyrin Conjugated Networks Achieves Energy‐Efficient Extraction of Uranium
Source: Adv Sci (Weinh). 2024 Oct 7;11(44):2409084. doi: 10.1002/advs.202409084 (PMC11600223; doi:10.1002/advs.202409084)
Supplement: Supplementary file 1 — Supporting Information [file ADVS-11-2409084-s002.docx]

Supporting Information

**Precise Electrocatalysis on Fe-porphyrin Conjugated Networks Achieves Energy-Efficient Extraction of Uranium**

*Wenwen Wang ^†^, Meiyun Xu ^†^, Haotian Wu, Yan Song, Peng Liu, Haisheng Yu, Linjuan Zhang, Shusen Chen ^*^, Daoben Hua ^*^*

† W. Wang and M. Xu contributed equally to this work.

W. Wang, M. Xu, P. Liu, D. Hua

State Key Laboratory of Radiation Medicine and Protection, School for Radiological and Interdisciplinary Sciences (RAD−X), Collaborative Innovation Center of Radiological Medicine of Jiangsu Higher Education Institutions, Soochow University, Suzhou 215123, China.

E-mail: dbhua_lab@suda.edu.cn

H. Wu, Y. Song, S. Chen

Beijing Research Institute of Chemical Engineering and Metallurgy, CNNC Key Laboratory on Uranium Extraction from Seawater, Beijing, China

E-mail: samCSS@163.com

H. Yu, L. Zhang

Key Laboratory of Interfacial Physics and Technology, Shanghai Institute of Applied Physics, Chinese Academy of Sciences, Shanghai 201800, China.

**Table of contents**

1. Materials and characterization......................................................................................................4

1.1. Materials....................................................................................................................................4

1.2. Preparation of modified electrodes............................................................................................4

1.3. Characterization........................................................................................................................4

2. Physicochemical sorption experiments........................................................................................5

2.1. Effect of pH value......................................................................................................................5

2.2. Investigation on sorption kinetics and isotherm........................................................................5

2.3. Adsorption Selectivity................................................................................................................6

2.4. Adsorption of U(Ⅵ) in simulated wastewater............................................................................7

3. Electrochemical extraction of uranium.........................................................................................7

3.1. The effect of voltage.................................................................................................................7

3.2. The effects of initial uranium concentration..............................................................................7

3.3. Electrochemical extraction in uranium-containing groundwater...............................................7

3.4. Sustained electrochemical extraction of uranium.....................................................................8

3.5. Uranium extraction from seawater............................................................................................8

4. Computational details..................................................................................................................8

Table S1...........................................................................................................................................9

Table S2...........................................................................................................................................9

Table S3...........................................................................................................................................9

Table S4..........................................................................................................................................10

Table S5..........................................................................................................................................10

Table S6..........................................................................................................................................11

Table S7..........................................................................................................................................11

Table S8..........................................................................................................................................12

Table S9..........................................................................................................................................13

Table S10........................................................................................................................................14

Table S11........................................................................................................................................15

Table S12........................................................................................................................................15

Table S13........................................................................................................................................16

Table S14........................................................................................................................................16

Table S15........................................................................................................................................17

Figure S1........................................................................................................................................18

Figure S2........................................................................................................................................18

Figure S3........................................................................................................................................19

Figure S4........................................................................................................................................19

Figure S5........................................................................................................................................20

Figure S6........................................................................................................................................20

Figure S7........................................................................................................................................21

Figure S8........................................................................................................................................21

Figure S9........................................................................................................................................22

Figure S10......................................................................................................................................22

Figure S11......................................................................................................................................23

Figure S12......................................................................................................................................23

Figure S13......................................................................................................................................24

Figure S14......................................................................................................................................24

Figure S15......................................................................................................................................25

Figure S16......................................................................................................................................25

Figure S17......................................................................................................................................26

Figure S18......................................................................................................................................26

Figure S19......................................................................................................................................27

Figure S20......................................................................................................................................27

Figure S21......................................................................................................................................28

Figure S22......................................................................................................................................28

Figure S23......................................................................................................................................29

Reference.......................................................................................................................................29

# 1. Materials and characterization

## 1.1. Materials

5,10,15,20-Tetrakis(4-aminophenyl)-21H,23H-porphine (TAPP) and 1,10-Phenanthroline-2,9-dicarboxylic acid (H_2_PDA) was purchased from Sinopharm Chemical Reagent Co. UO_2_(NO_3_)_2_·6H_2_O Fe foil, Fe_4_Nd_2_O_9_, Fe_2_O_3_ and SrFeO_3_ were purchased from Aladdin, 99.7% purity. The U stock solution was prepared by dissolving the desired amount of UO_2_(NO_3_)_2_·6H_2_O (99%) solid in deionized water. NaCl, NaOH, NaNO_3_, Na_2_SO_4_, FeSO_4_ and other reagents were analytically pure and used as received. Deionized water (18.2 MΩ·cm) was used for all experiments.

## 1.2. Preparation of modified electrodes

Fe@PDACN (50 mg) and multi-walled carbon nanotubes (conductive additive, 6 mg) were dispersed in 1.3 mL of ethanol (containing 100 µL of 20.0 wt.% Nafion solution) and sonicated for 30 min to form an ink. The density of 20.0 wt.% Nafion solution is 0.206 g mL^-1^, so the content of adsorbent in the ink is 65.3 wt.% (except ethanol). Then 60 µL of ink was dropped onto 1 x 1.2 cm^2^ super-hydrophilic conductive carbon cloth with a pipette gun and dried in a moisture-proof cabinet. The loading-content of the adsorbent on the electrode was obtained by multiplying the mass difference before and after loading by 65.3%.

## 1.3. Characterization

The solid-state ^13^C NMR spectroscopy was carried out on a Bruker Avance Ⅲ model 400 MHz NMR spectrometer at a MAS rate of 5 kHz. Fourier transform infrared spectroscopy (FTIR) spectra of materials in the 4000-400 cm^-1^ range were collected by a Thermo Nicolet iS50 spectrometer. SEM and energy-dispersive spectroscopy (EDS) images were acquired with the energy of the electron beam being 20 keV using a FEI Quanta 200FEG scanning electron microscope (SEM). The morphology and elemental mapping were recorded by a FEI Tecnai G2 field emission high-resolution transmission electron microscope (HRTEM) with an accelerating voltage of 200 kV. X-ray photoelectron spectroscopy (XPS) was carried out by an ESCALAB 250Xi spectrometer. X-ray diffraction (XRD) pattern of PDACN and Fe@PDACN were analyzed using a Bruker D8 Advance diffractometer with Cu Kα radiation and a Lynxeye 1D detector, with a test angle range from 5° to 70° and a gradient of 0.02°. Surface areas and pore size distributions were measured by N_2_ sorption and desorption at 77.3 K using the ASAP 2020 volumetric sorption analyzer. BET surface areas were calculated over the relative pressure range 0.05-0.15 P/P_0_. Samples were degassed at 100 ℃ for 10 h under high vacuum before analysis. The U L_3_-edge and Fe K-edge X-ray absorption spectroscopy were collected in the transmission mode using inhouse laboratory-based X-ray absorption spectrometer. All XAFS data were analyzed using the program Demeter. For all samples, the EXAFS oscillations were extracted from the normalized XAS spectra by subtracting the atomic background using a cubic spline fit to k^3^-weighted data, where k is the photoelectron wave number. The χ(k) functions were then Fourier transformed into R-space.

# 2. Physicochemical sorption experiments

A typical adsorption procedure was as follows: the adsorbent was added into a centrifuge tube containing uranium solutions with varying concentrations. The dosage of adsorbent and the adsorption time were arranged according to the specific experiments. After stirring for a desired period of time at 120 rpm on an incubator shaker at 25 ℃, the mixture was filtered with a syringe filter (Nylon−66, 0.22 μm). The concentration of uranium was determined using ICP−OES. The equations for sorption efficiency (SE, %), and adsorption capacity (*q*, mg g^-1^) are as formula S1 and formula S2:

$\mathrm{SE} \left( \% \right) = \frac{C_{0}-C_{e}}{C_{0}} \times100\%$ (S1)

$q=(C_{0}-C_{e})\frac{V}{m}$ (S2)

where *C*_0_ (mg L^-1^) is the initial uranium concentration, *C*_e_ (mg L^-1^) is the equilibrium U concentration, *V* (L) is the volume of the test solution, and m (g) is the mass of the adsorbent.

## 2.1. Effect of pH value

The effect of pH values in the range of 1 to 8 on uranium sorption was investigated. Adjust the desired pH with NaOH and HNO_3_ solution. 5 mg of adsorbents (PDACN or Fe@PDACN) was added to 50 mL of aqueous solution containing 11.9 mg L^-1^ uranium and shaken for 3 h at 25 ℃ in a thermostat.

## 2.2. Investigation on sorption kinetics and isotherm

Adsorption kinetics experiments of U were carried out at pH 4.0±0.1. 5 mg of adsorbents (PDACN or Fe@PDACN) was added to 50 mL of aqueous solution containing 11.9 mg L^-1^ uranium and shaken at 25 ℃ in a thermostat. Samples were collected at different times.

Pseudo-first-order model (formula S3) and pseudo-second-order model (formula S4) were used to fit the adsorption kinetic data. The equations for these two models are expressed as follows^[1]^:

$\log\left( q_{e}-q_{t} \right)=\log q_{e}-\frac{k_{1}}{2.303}t$ (S3)

$\frac{t}{q_{t}}=\frac{1}{k_{2}q_{e}^{2}}+\frac{t}{q_{e}}$ (S4)

where *q*_t_ (mg g^-1^) and *q*_e_ (mg g^-1^) are the adsorption capacity at the contact time *t* and adsorption equilibrium, respectively. *k*_1_ (min^-1^) and *k*_2_ (g mg^-1^ min^-1^) are the pseudo-first-order and pseudo-second-order rate constants, respectively.

Adsorption isotherm experiments were performed at pH 4.0±0.1 by varying the initial concentration of U. 5 mg of adsorbents (PDACN or Fe@PDACN) were added to 50 mL of aqueous solution containing uranium and shaken for 3 h at 25 ℃ in a thermostat. The final uranium content was determined using ICP−OES.

The Langmuir isotherm is based on the assumption of monolayer adsorption with uniform surface properties of the adsorbent, and the linear equation is expressed as formula S5^[2]^:

$\frac{C_{e}}{q_{e}}=\frac{1}{q_{\max}K_{L}}+\frac{C_{e}}{q_{\max}}$ (S5)

where *q*_max_ (mg g^-1^) is the maximum adsorption capacity. *K*_L_ (L mg^-1^) is a constant of the Langmuir model, which characterizes the affinity of the adsorbate to the adsorbent.

The Freundlich equation is based on heterogeneous surface adsorption. Adsorbates occupy stronger binding sites first, and the binding strength decreases with the increase of site occupation. The linear equation can be expressed as formula S6:

$\ln q_{e}={\ln K}_{F}+\frac{1}{n}\ln C_{e}$ (S6)

where *K*_F_ (mol^1-n^ L^n^ g^-1^) and n are the Freundlich constants related to adsorption capacity and adsorption strength, respectively.

## 2.3. Adsorption Selectivity

The effect of coexisting ions on adsorption was carried out in a multicomponent cation solution at pH 4.0±0.1. The concentration of each ion was 1 × 10^-4^ mol L^-1^, including alkali metals (Li^+^, Rb^+^), alkaline earth metals (Mg^2+^, Sr^2+^), transition metals (Fe^3+^, Co^2+^, Zn^2+^, Cu^2+^), and lanthanides (Sm^3+^, La^3+^). 5 mg of Fe@PDACN was added to 50 mL of mixed ionic solution and shaken in a thermostat at 25 ℃ for 3 h. The concentrations of uranium and other ions in the solutions before and after adsorption were measured using ICP-OES.

The partition coefficient (*K*_d_) of Fe@PDACN toward uranium can be calculated according to formula S7:

$K_{d}=\frac{{(C}_{0}-C_{e})V}{mC_{e}}$ (S7)

where *C*_0_ (mg L^-1^) is the initial uranium concentration, *C*_e_ (mg L^-1^) is the equilibrium uranium concentration, *V* (L) is the volume of the test solution, and *m* (g) is the mass of the adsorbent

The salt resistance was investigated at pH 4.0 ± 0.1 by selecting NaCl, Na_2_SO_4_ and NaNO_3_. The salt content was 0.5 mol L^-1^ and 1 mol L^-1^, and the uranium content was 50 mg L^-1^. 5 mg of Fe@PDACN was added to 5 mL of solution and shaken for 3 h at 25 ℃ in a thermostat.

## 2.4. Adsorption of U(VI) in simulated wastewater

The uranium-containing wastewater, including simulated nuclear wastewater (Table S6)^[3]^ and simulated uranium-containing groundwater (Table S7),^[4]^ was prepared according to the scheme reported in the literature. 5 mg of adsorbent was added to 5 mL of solution and shaken for 3 h at 25 ℃ in a thermostat.

# 3. Electrochemical extraction of uranium

## 3.1. The effect of voltage

The performance of electrochemical uranium extraction at different constant voltages (0 V, -0.6 V, -1.2 V, -1.5 V, -1.8 V) was investigated. A solution of uranium with a concentration of 100 mg L^-1^ was added to the electrochemical cell resulting in a solid-liquid ratio of 0.1 g L^-1^. The solution samples were collected at different times.

## 3.2. The effects of initial uranium concentration

Electrochemical uranium extraction experiments with different initial uranium concentrations were carried out at 25 ℃ and -1.5 V constant voltage. The initial uranium concentrations were 100 mg L^-1^, 500 mg L^-1^, 1000 mg L^-1^, 1500 mg L^-1^, and 2000 mg L^-1^, respectively, and the solid-liquid ratio was 0.1 g L^-1^. The solution samples were collected at different times.

## 3.3. Electrochemical extraction in uranium-containing groundwater

The simulated uranium-containing groundwater was formulated based on reports of uranium-bearing groundwater (Table S7).^[4]^ The solid-liquid ratio was set at 0.0015 g L^-1^ (3 mg/2000 mL). At increasing time intervals, 3 mL aliquots were removed from the mixture, filtered through a 0.45 μm membrane filter.

## 3.4. Sustained electrochemical extraction of uranium

Sustained electrochemical extraction of uranium without elution was carried out in simulated nuclear wastewater (Table S6) at a solid-liquid ratio of 0.02 g L^-1^ (4 mg/200 mL). The electrochemical reaction cell is a 250 mL beaker. Using two modified electrodes as working electrodes, the total adsorbent loading was controlled to 4 ± 0.1 mg. There was no need to elute after each electrochemical uranium extraction, and the solution was changed directly for the next electrochemical uranium extraction. The solution samples were collected at different times.

## 3.5 Uranium extraction from seawater

Seawater is filtered through a 0.25 μm membrane filter and used. The pH of seawater is determined to be 8.6. Use of a square wave with a frequency of 1 Hz and a voltage of 1.5 V/0 V for the electrochemical extraction of uranium from seawater. Firstly, the extraction efficiency of uranium was tested in seawater doped with 1 mg L^-1^ uranium at a solid-liquid ratio of 0.01 g L^-1^ (2 mg/200 mL). At increasing time intervals, 1 mL aliquots were removed from the mixture, filtered through a 0.45 μm membrane filter. The filtrates were diluted, and analyzed by ICP−MS. Electrochemical uranium extraction from seawater was carried out for 48 h at a solid-liquid ratio of 0.0005 g L^-1^ (2 mg/4000 mL). The electrodes were digested in aqua regia after electrochemical extraction, and the digested solution was volume fixed and diluted. The diluted solution was analyzed by ICP−MS.

# 4. Computational details

Density functional theory (DFT) calculations were performed using the Gaussian 09 program. All ion and complex geometries were optimized at B3LYP−D3/SDD~6−31G* level.^[5]^ Grimme’s empirical dispersion (GD3) correction was used to improve the description of van der Waals interactions^[5b]^. The Stuttgart/Dresden relativistic effective core potentials (SDD) and corresponding valence basis sets were applied for the Fe atom and uranium atom.^[5d]^ For single−point energy calculations, higher precision calculations were performed on these optimized−structures at the PBE0-D3/SDD~6−31+G* level.^[5a, 5d, 6]^ The SMD implicit solvent model was used to consider the solvent (water) effects.^[6a]^ Each optimized structure was confirmed with all real frequencies, and the zero-point corrections were counted. It should be noted that for Fe(Ⅱ), the high spin state complexes (quintet state) are more stable than the low spin state complexes (singlet state).^[7]^

| **Table S1.** Parameters of Fe K-edge EXAFS curve fitting for Fe@PDACN.^*^ | | | | | | |
| --- | --- | --- | --- | --- | --- | --- |
| Sample | shell | C.N. | R(Å) | σ^2^(Å^2^) | *Δ*E (eV) | R−factor |
| Fe@PDACN | Fe−N | 4.25 | 2.06(2) | 0.005(2) | 6.82(0) | 0.0091 |

^*^ T = 298 K. C.N. means coordination number of the neighbors;

R (Å) is the bond distance, and σ^2^ is the Debye−Waller factor;

*Δ*E (eV) is Energy shift relative to the calculated Fermi level;

R−factor is Goodness−of−fit indicator.

| **Table S2.** Specific surface area and porosity parameters of PDACN and Fe@PDACN. | | | |
| --- | --- | --- | --- |
| Material | Specific surface areas (m^2^ g^-1^) | Pore volume (cm^3^ g^-1^) | Average pore diameter (nm) |
| PDACN | 8.0 | 0.060 | 9.6 |
| Fe@PDACN | 10.7 | 0.058 | 8.8 |

| **Table S3.** Kinetic parameters for the adsorption of U(Ⅵ) by PDACN and Fe@PDACN.^*^ | | | | | | | |
| --- | --- | --- | --- | --- | --- | --- | --- |
| Sorbent | *q*_e,exp_  (mg g^-1^) | pseudo-first-order | | | pseudo-second-order | | |
|  |  | *q*_e,cal_  (mg g^-1^) | *k*_1_  (min^-1^) | R^2^ | *q*_e,cal_  (mg g^-1^) | *k*_2_  (g min^-1^ mg^-1^) | R^2^ |
| PDACN | 100.24 | 3.29 | 0.291 | 0.663 | 98.52 | 0.138 | 0.999 |
| Fe@PDACN | 102.14 | 1.89 | 0.229 | 0.406 | 100.60 | 0.122 | 0.999 |

^*^ Conditions: solid-liquid ratio = 0.1 g L^-1^, *C*_U(Ⅵ)_ = 11.9 mg L^-1^, pH 4.0 ± 0.1, and 298 K

| **Table S4.** Langmuir and Freundlich parameters for the adsorption of U(Ⅵ) by PDACN and Fe@PDACN. | | | | | | |
| --- | --- | --- | --- | --- | --- | --- |
| Sorbent | Langmuir | | | Freundlich | | |
|  | *q*_max_ (mg g^-1^) | *K*_L_ (L mg^-1^) | R^2^ | *K*_F_ (L g^-1^) | n | R^2^ |
| PDACN | 376.37 | 1.146 | 0.999 | 136.90 | 3.137 | 0.711 |
| Fe@PDACN | 384.01 | 1.287 | 0.997 | 158.88 | 3.444 | 0.710 |

Conditions: solid-liquid ratio = 0.1 g L^-1^, *C*_U(Ⅵ)_ = 10 ~ 100 mg L^-1^, pH 4.0 ± 0.1, and 298 K

| **Table S5.**  Comparison of sorption capacity (*q*_e_) for PDACN and Fe@PDACN with that of other materials. | | |
| --- | --- | --- |
| Adsorbent | *q*_e_ (mg g^-1^) | Reference |
| 0.4-PEI/ECH-CTS | 380.65 | [8] |
| NH_2_-UiO-66/g-C_3_N_4_ | 195.6 | [9] |
| Graphene aerogel | 238.67 | [10] |
| CMP-OC | 197.6 | [11] |
| t-DOPOR | 69.49 | [12] |
| (PHMB-GO)0.4@Gelatin-PAM | 625.00 | [13] |
| HCPA-2 | 27.7 | [14] |
| Ni/Al-LDHs | 159.7 | [15] |
| CTHM-2 | 580 | [16] |
| Si/UiO-66-AO | 217 | [17] |
| PCF-20-600 | 1250 | [18] |
| TzDa-Phos | 394 | [19] |
| ITDCN-1 | 1365.7 | [20] |
| **PDACN** | **376.37** | **This work** |
| **Fe@PDACN** | **384.01** | **This work** |

| **Table S6.** Constituents of simulated nuclear wastewater.^[3]^ | |
| --- | --- |
| Constituents | Concentration (mg L^-1^) |
| U(Ⅵ) | 50.0 |
| Sr^2+^ | 98.6 |
| Ca^2+^ | 50.0 |
| Mg^2+^ | 50.0 |
| Na^+^ | 96358.3 |
| Cs^+^ | 157.9 |
| Cl^-^ | 35500 |
| NO_3_^-^ | 186000 |
| SO_4_^2-^ | 9600 |

| **Table S7** Constituents of simulated uranium-containing groundwater.^[4]^ | |
| --- | --- |
| Constituents | Concentration (mg L^-1^) |
| U(Ⅵ) | 1.38 |
| Cl^-^ | 35000 |
| HCO_3_^-^ | 586.82 |
| Na^+^ | 175.26 |
| Mg^2+^ | 266.88 |
| K^+^ | 14.82 |
| Ca^2+^ | 88.8 |

| **Table S8.** Comparison of distribution coefficient (*K*_d, U_) for Fe@PDACN with that of other materials. | | |
| --- | --- | --- |
| Adsorbent | *K*_d,U_ (mL g^-1^) | Reference |
| 0.4-PEI/ECH-CTS | 1.1×10^4^ | [8] |
| MIGPAF-13 | 2.0×10^6^ | [21] |
| NHPC | 1.62×10^3^ | [22] |
| t-DOPOR | 2.54×10^4^ | [12] |
| (PHMB-GO)0.4@Gelatin-PAM | 1.73×10^4^ | [13] |
| HCPA-2 | 6.95×10^2^ | [14] |
| AO-Imp(250) fiber | 3.20×10^3^ | [23] |
| PACNC | 5.85×10^4^ | [24] |
| PCF-20-600 | 1.5×10^4^ | [18] |
| TzDa-Phos | 5.8×10^3^ | [19] |
| ITDCN-1 | 9.65×10^5^ | [20] |
| **Fe@PDACN** | **2.79×10^5^** | **This work** |

| **Table S9.** Distribution ratio *K*_d_ and selectivity coefficients *β* of Fe@PDACN. | | |
| --- | --- | --- |
| Metal ion | *K*_d_ (mL g^-1^) | *β* (*K*_d_, U/*K*_d_, ions) |
| U(Ⅵ) | 278926.09 | -- |
| Li^+^ | 361.77 | 966.89 |
| Rb^+^ | 147.03 | 2556.20 |
| Mg^2+^ | 810.64 | 577.00 |
| Sr^2+^ | 335.28 | 962.03 |
| Fe^3+^ | 2761.81 | 108.99 |
| Co^2+^ | 1482.67 | 195.26 |
| Zn^2+^ | 949.53 | 374.23 |
| Cu^2+^ | 939.85 | 319.59 |
| Sm^3+^ | 448.22 | 772.93 |
| La^3+^ | 386.15 | 674.67 |

| **Table S10.**  Comparison of electrochemical uranium extraction performance of different electrode materials. | | | |
| --- | --- | --- | --- |
| Materials | Experimental conditions | Performance | Reference |
| Fe−N_x_−C−R | square wave voltage of -5 V/0 V, [U]_0_ = 2000 mg L^-1^ | 14302 mg g^-1^ | [25] |
| C-Ami electrode | square wave voltage of -5 V/0 V, [U]_0_ = 2000 mg L^-1^ | 1932 mg g^-1^ | [26] |
| TETA-PAO/GF | square wave voltage of -3 V/0 V, [U]_0_ = 1000 mg L^-1^ | 1142 mg g^-1^ | [27] |
| LIG6/Co_4_S_3_-15 | 1.2 V constant potential, Langmuir isotherm fitting | 2703 mg g^-1^ | [28] |
| HGNbP | constant voltage of -1.2 V, [U]_0_ = 1000 mg L^-1^ | 1340 mg g^-1^ | [29] |
| CSKN-15 | constant voltage of -1.2 V, [U]_0_ = 360 mg L^-1^ | 443 mg g^-1^ | [30] |
| BC/PPy | constant voltage of -0.9 V, [U]_0_ = 100 mg L^-1^ | 237.9 mg g^-1^ | [31] |
| 3D-FrGOF | −0.9 V vs SCE, [U]_0_ = 600 mg L^-1^ | 4560 mg g^-1^ | [32] |
| MIGPAF-13 | constant voltage of -1.3 V, [U]_0_ = 8 mg L^-1^ | 419.2 mg g^-1^ | [21] |
| **Fe@PDACN** | **constant voltage of -1.5 V, [U]_0_ = 50 mg L^-1^** | **24646.3 mg g^-1^** | **This work** |

| **Table S11**. Energy cost and coulombic efficiency of Fe@PDACN based sustained electrochemical extraction.^*^ | | | | | |
| --- | --- | --- | --- | --- | --- |
| U (V) | I (A) | *m*_U_ (g) | Energy consumption (kWh kg^-1^ U) | Energy cost ($ kg^-1^ U) | Coulombic efficiency (%) |
| -1.5 | 8.8 × 10^−5^ | 9.86 × 10^−2^ | 0.375 | 3.22 × 10^−2^ | 90.1 |
| ^*^*m*_U_ (g) is the total mass of uranium extracted in 10 times. The mass of uranium extracted each time (kg) = *V*(*C_0_*-*C_e_*) × 10^−3^, where *V* (L) is the volume of wastewater, *C_0_* (mg L^-1^) and *C_e_* (mg L^-1^) are the initial and final concentrations of uranium, respectively. Energy consumption (kWh kg^-1^ U) = *UIt*/*m*_U_, where *U* (V) and *I* (A) are voltage and average current respectively. *t* (h) is the total time for 10 extractions. The price of electricity used was ~8.6 ¢/kWh (Shanghai commercial average electricity prices in 2023 from State Grid Corporation of China). The energy cost is calculated as 3.22 × 10^−2^ $ kg^−1^ U. Coulombic efficiency (%) = 100 × z*N_A_m*_U_e/*MIt*, where z is the stoichiometric coefficient of electron transfer for electrochemical extraction of uranium (z = 2). *N_A_* is Avogadro constant (*N_A_* = 6.02 × 10^23^ mol^-1^) and e is elementary charge (e = 1.602 × 10^-19^ C). *M* is the relative mass of U-238 (M = 238 g mol^-1^). | | | | | |

| **Table S12.** Comparison of energy consumption and energy costs for electrochemical U(Ⅵ) extraction based different materials. | | | |
| --- | --- | --- | --- |
| Materials | Energy consumption (kWh kg^-1^ U) | Energy cost ($ kg^-1^ U) | Reference |
| C-Ami electrode | -- | 1900 | [26] |
| Ti foil | -- | 0.55~64.65 | [33] |
| In–N_x_–C–R | -- | 806 | [34] |
| PA-PANI/GS | -- | 632 | [35] |
| CF | 10.63 | -- | [36] |
| CF-AO | 6.52 | -- | [36] |
| MoS_2_ nanosheets | 294.9 | -- | [37] |
| **Fe@PDACN** | **0.375** | **3.22 × 10^-2^** | **This work** |

| **Table S13.** Comparison of the uranium extraction capacities from natural seawater of Fe@PDACN with other materials. | | | | |
| --- | --- | --- | --- | --- |
| Adsorbent | Adsorption capacity | | | Reference |
|  | mg g^-1^ | Time | Conditions |  |
| BSA@CFF | 6.6 | 18 days | -2.6 V; 10 mg/20 L | [38] |
| Fe–N_x_–C–R | 1.2 | 1 day | -5 V~0 V square wave; 6 mg/2.4 L | [25] |
| B: Cu-PO_4_ | 0.71 | 8 hours | -1.7 V; 35 mg/10 L | [39] |
| TETA-PAO/GF | 0.46 | 20 days | -3 V~0 V square wave; 103 mg/16 L | [27] |
| MIGPAF-13 | 16.0 | 56 days | -1.3 V | [21] |
| PPA@MISS-PAF-1 | 13 | 56 days | -1.3 V~0 V square wave | [40] |
| PAO-G-A | 1.14 | 40 days | kg-grade marine tests | [41] |
| PAO-PNM | 9.35 | 35 days | 36 mg/1000 L; flow rate = 90 L/h | [42] |
| PAO-Co | 9.7 | 49 days | 10 mg/100 L | [43] |
| VA-PG | 13.63 | 32 days | 100 mg/100 L; flow rate = 600 L/h | [44] |
| MUU | 7.35 | 16 days | 8 mg/100 L; flow rate = 60 L/h | [45] |
| AO-OpNpNc fibers | 17.57 | 90 days | 1 kg in ocean | [46] |
| Zn^2+^–PAO | 9.23 | 28 days | V = 1 L; flow rate = 90 L/h | [47] |
| NDA-TN-AO | 6.07 | 27 days | 10 mg/100 L | [48] |
| UiO-66-AO | 2.68 | 3 days | 1 mg/1 L | [49] |
| H-ABP fiber | 11.5 | 90 days | kg-grade marine tests | [50] |
| **Fe@PDACN** | **4.8** | **2 days** | **-1.5 V~0 V square wave; 2 mg/4 L** | **This work** |

| **Table S14.** Parameters of U L-edge EXAFS curve fitting for Fe@PDACN after physicochemical adsorption of uranyl (Fe@PDACN-U).^*^ | | | | | | |
| --- | --- | --- | --- | --- | --- | --- |
| Sample | shell | C.N. | R(Å) | σ^2^(Å^2^) | *Δ*E(eV) | R−factor |
| Fe@PDACN-U | U=O_uranyl_ | 1.91 | 1.76(2) | 0.003(5) | 10.8(7) | 0.008 |
|  | U−O_w_ | 2.23 | 2.35(6) | 0.006(7) |  |  |
|  | U−O_amide_(N_PDA_) | 3.77 | 2.27(3) | 0.007(3) |  |  |

^*^ T=298 K. C.N. means coordination number of the neighbors;

R(Å) is the bond distance, and σ^2^ is the Debye−Waller factor;

*Δ*E (eV) is Energy shift relative to the calculated Fermi level;

R−factor is Goodness−of−fit indicator.

| **Table S15.** Single point energy (E_b_) for optimized structures by DFT theoretical calculations.^*^ | | |
| --- | --- | --- |
| Item | Spin multiplicity | E_b_ (a.u.) |
| Fe(Ⅱ)@PDACN | **5** | **-1111.23807645** |
| Fe(Ⅲ)@PDACN | 4 | -1111.08168432 |
|  | **6** | **-1111.09774803** |
| Fe@PDACN-U | 1 | -1738.36245253 |
|  | **3** | **-1738.4098013** |
| UO_2_^2+^ | **1** | **-627.13480616** |
|  | 3 | -627.04399608 |
|  | 5 | -626.80414414 |
| UO_2_^+^ | **2** | **-627.32045793** |
|  | 4 | -627.20885293 |
|  | 6 | -626.96946 |

^*^ The adopted results are marked in bold.

All ion and complex geometries were optimized at B3LYP−D3/SDD~6−31G* level.^[5]^

For single−point energy calculations, higher precision calculations were performed on these optimized−structures at the PBE0-D3/SDD~6−31+G* level.


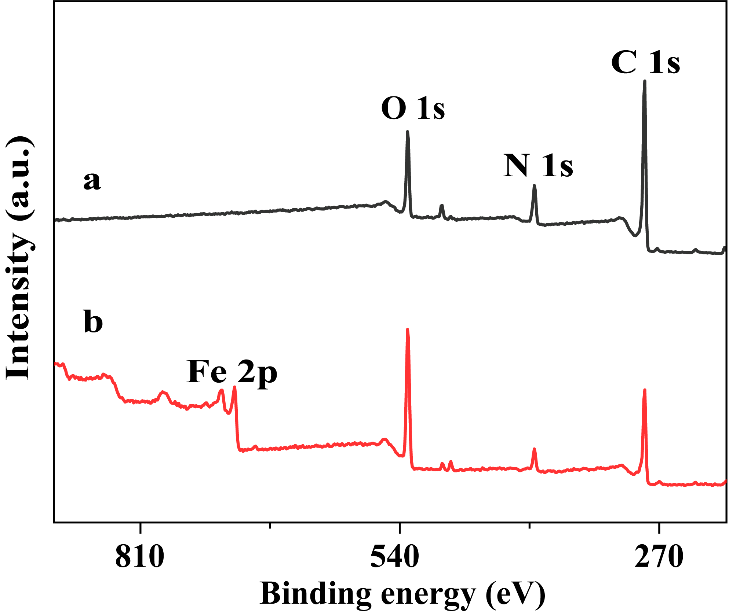


Figure S1. XPS survey spectra of PDACN and Fe@PDACN (a: PDACN, b: Fe@PDACN).


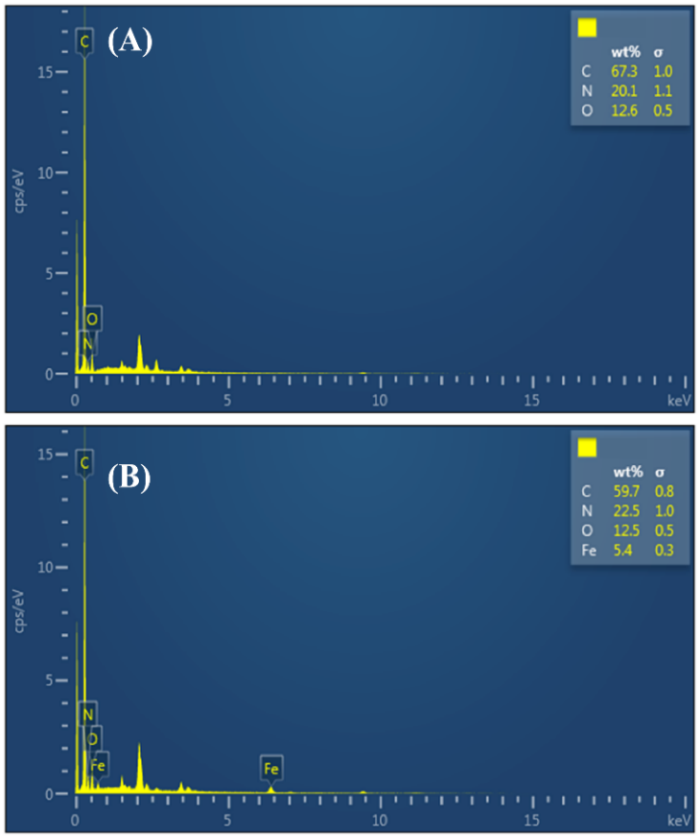


Figure S2. EDS spectra of A) PDACN and B) Fe@PDACN.


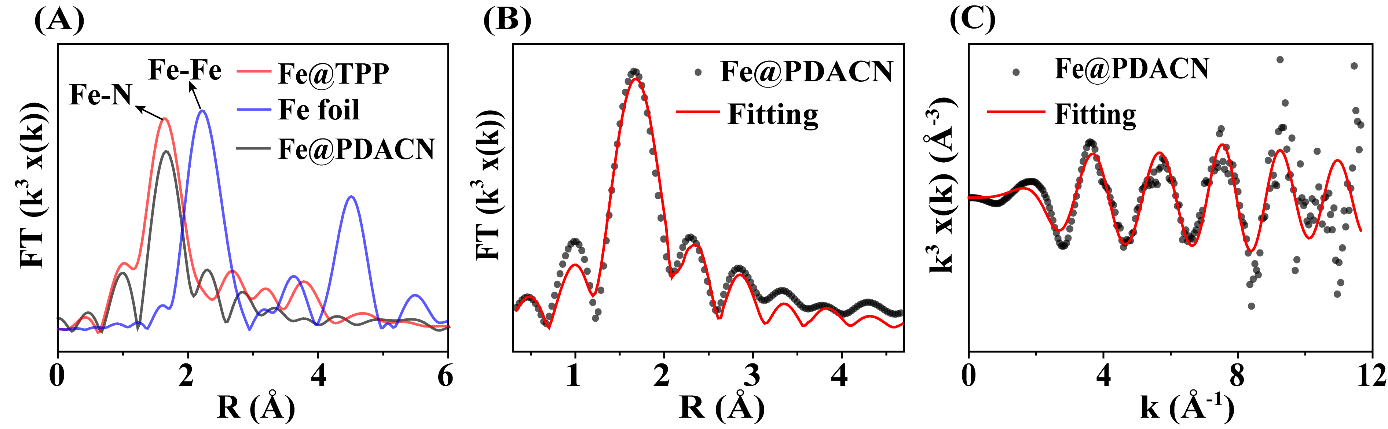


Figure S3. A) Fourier transform (FT) EXAFS spectra for Fe@PDACN, Fe@TPP, and Fe foil. B) Corresponding EXAFS R-space fitting curves for Fe@PDACN. C) Corresponding EXAFS k space fitting plots of Fe@PDACN. k-range of 3−9 Å^−1^, R range 1−3 Å, s0^2^ 0.90. (The FT-EXAFS spectra of Fe@PDACN shows a sharp peak of about 1.5 Å in R-space (Figure S3A). This is similar to Fe-tetraphenylporphyrin (Fe@TPP, Aladdin, 99.9% purity) which contains Fe-porphyrin structure. The EXAFS fitting results show Fe-N coordination number is 4.25, further confirming that the Fe-porphyrin structures in Fe@PDACN, that has a Fe-N length of 2.06(2) Å (Figure S3B, C and Table S1)).


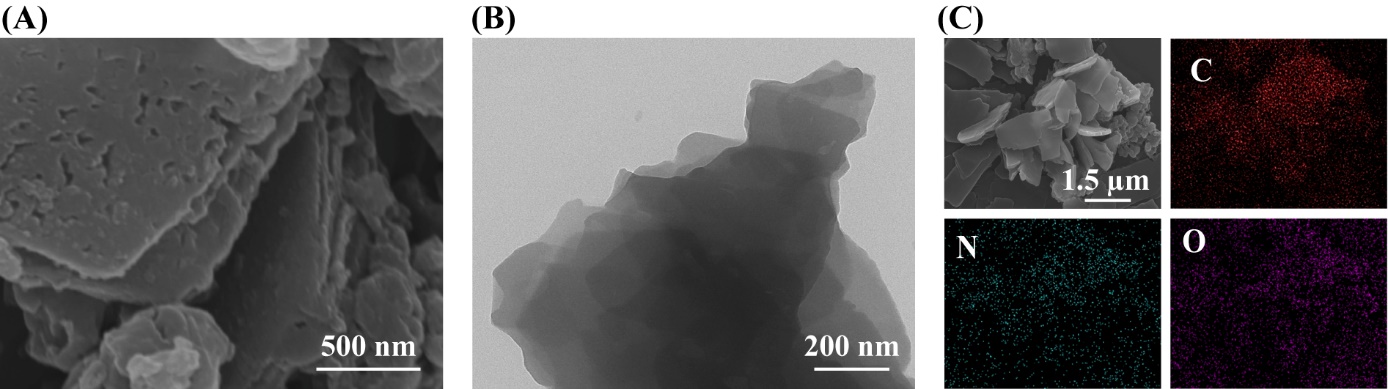


Figure S4. A) SEM image of PDACN. B) TEM image of PDACN. C) The EDS maps of PDACN for the elements C, N, and O.


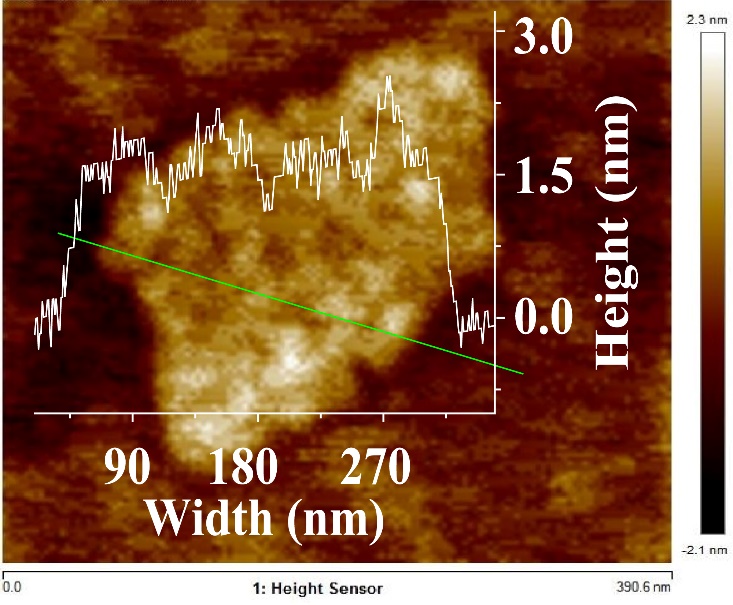


Figure S5. AFM height image of Fe@PDACN.


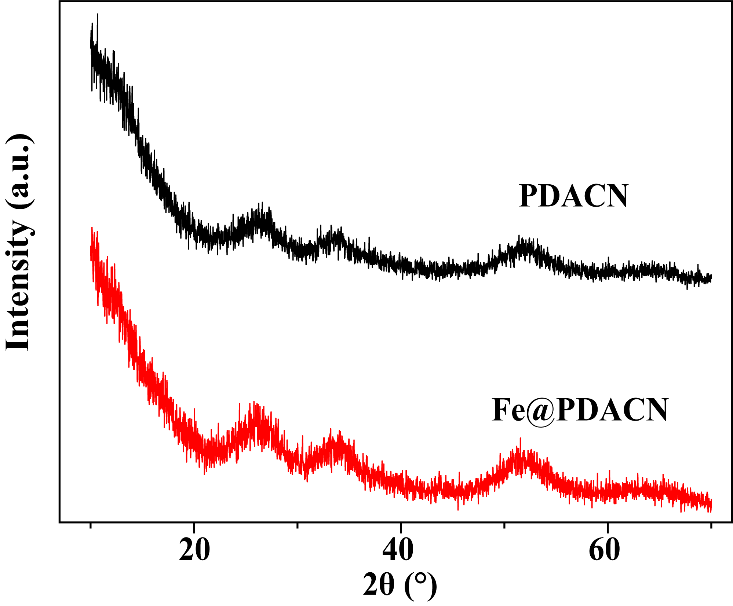


Figure S6. Powder X-ray scattering spectra of PDACN and Fe@PDACN. (PXRD patterns show PDACN and Fe@PDACN have broad peaks near 26°, 34°, and 52°, indicating a low crystallinity of materials.^[51]^)


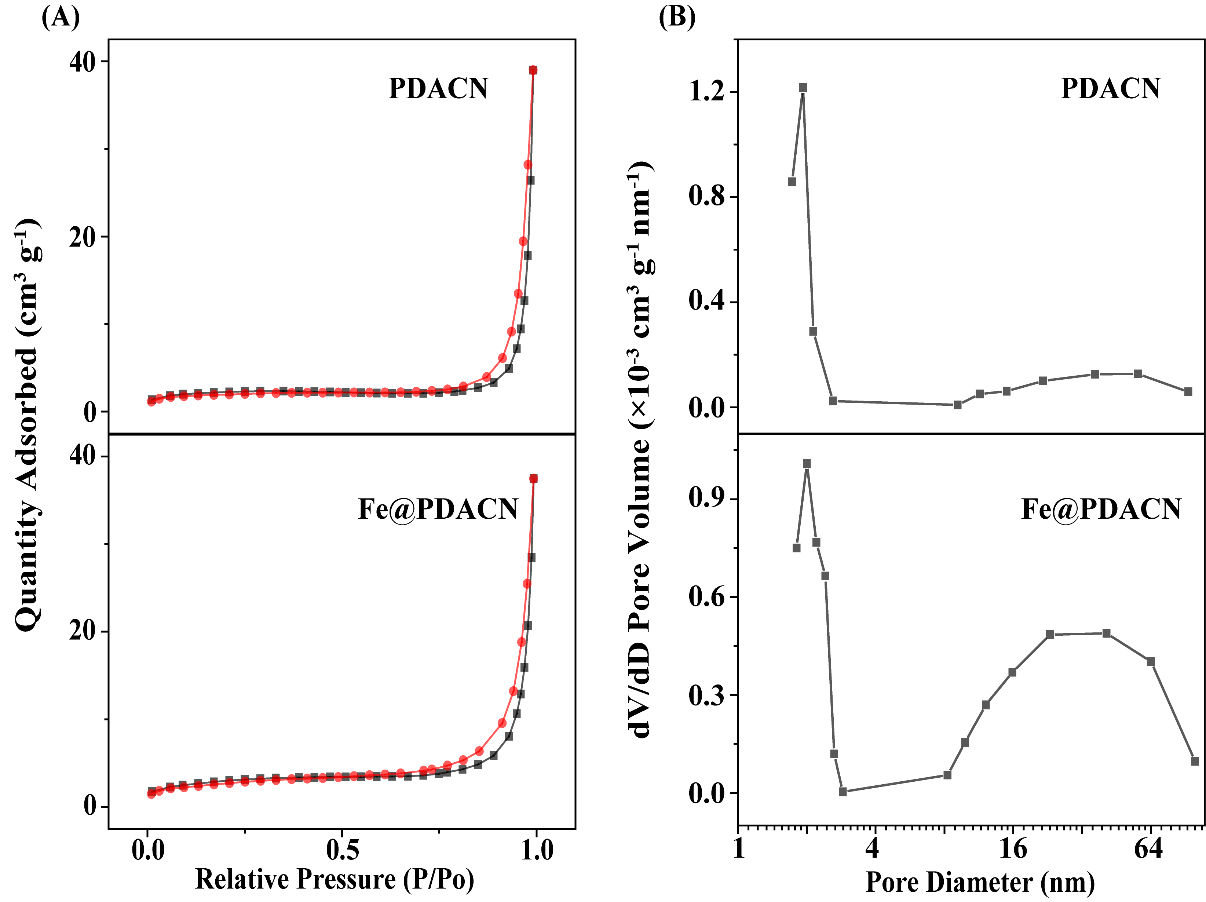


Figure S7. A) Nitrogen sorption/desorption isotherms and B) pore size distribution for PDACN and Fe@PDACN. (The specific surface areas of PDACN and Fe@PDACN determined by Brunner−Emmet−Teller (BET) measurements were 8.0 m^2^ g^-1^ and 10.7 m^2^ g^-1^, respectively (Table S2)).


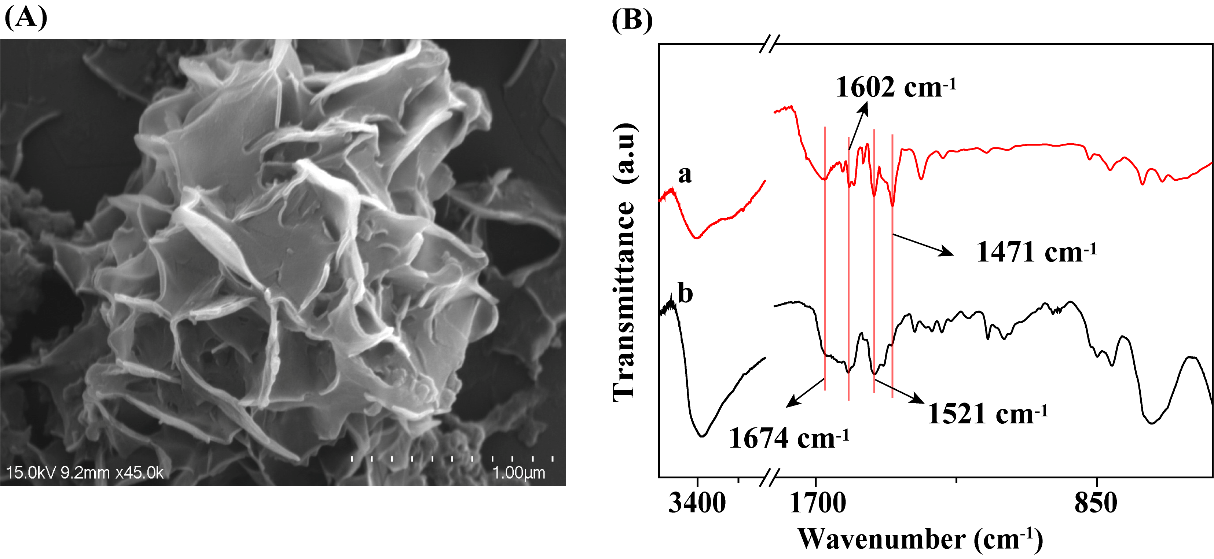


Figure S8. A) SEM images of Fe@PDACN after immersion in 1 M HNO_3_ for 24 h. B) FTIR spectra of Fe@PDACN and Fe@PDACN after immersion in 1 M HNO_3_ for 24 h (a: Fe@PDACN after immersion in 1 M HNO_3_ for 24 h; b: Fe@PDACN).


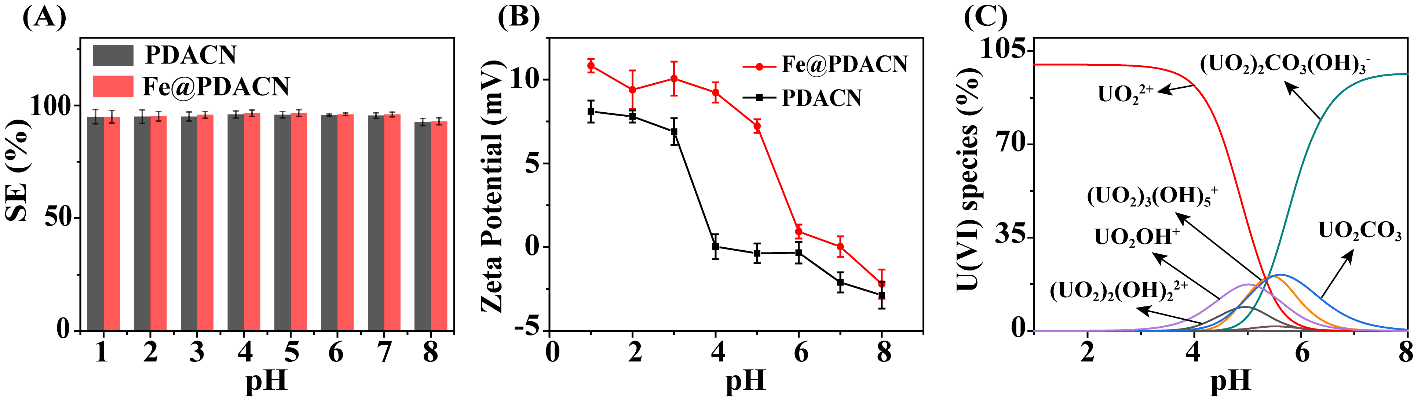


Figure S9. A) Effect of the pH value on U(Ⅵ) adsorption onto PDACN and Fe@PDACN. Conditions: Solid-liquid ratio = 0.1 g L^-1^, *C*_U(Ⅵ)_ = 11.9 mg L^-1^. B) Zeta potentials of PDACN and Fe@PDACN at different pH values. Conditions: Solid-liquid ratio = 0.1 g L^-1^. C) Distribution of U(Ⅵ) species as a function of pH in aqueous solution (Simulated by Visual MINTEQ, *C*_U(Ⅵ)_ = 11.9 mg L^-1^). (PDACN and Fe@PDACN maintain high adsorption efficiency (>90%) within the pH range of 1.0-8.0 (Figure S9A). Figures S9B and S9C show that in the pH ranges of 1.0-4.0 and 8.0, both PDACN and Fe@PDACN exhibit the same surface charge characteristics as the major uranium species, which typically leads to electrostatic repulsion between the adsorbate and the adsorbent. Therefore, electrostatic interactions did not play a positive role in the adsorption process. It was demonstrated that PDA binds hard-soft donors in the same molecule and its ability to complex uranyl ions usually plays a dominant role in adsorption.^[52]^ Thus, the high adsorption efficiency can be attributed to the high affinity of the PDA ligands for uranyl ions. Chemical coordination dominated the adsorption process, which was well supported by the results of the subsequent adsorption kinetic fitting (Figure 3A and Table S3).)


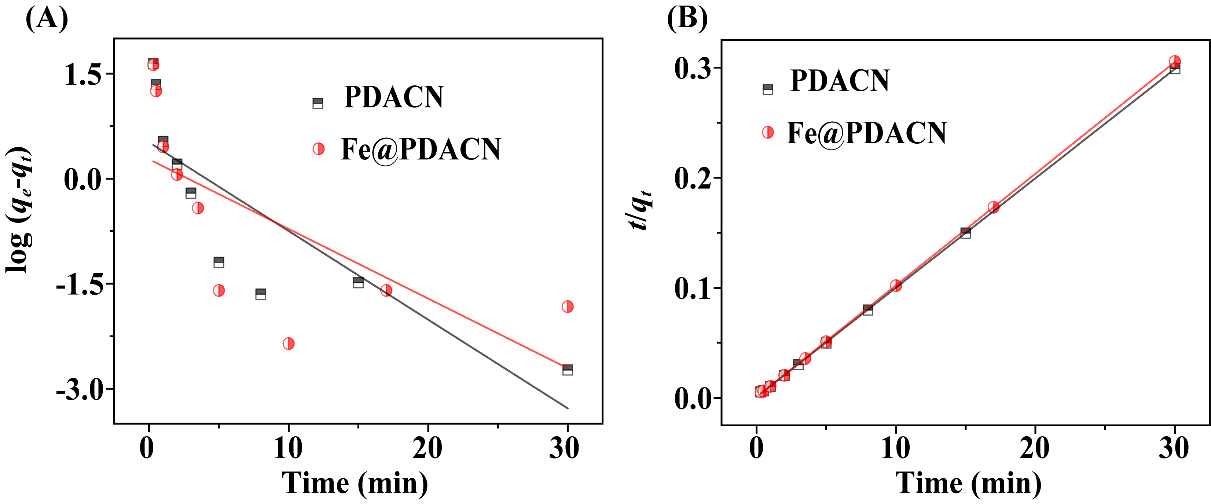


Figure S10. Linear regression of A) the pseudo-first-order and B) the pseudo-second-order kinetics models of U(Ⅵ) sorption on PDACN and Fe@PDACN (Conditions: solid-liquid ratio = 0.1 g L^-1^, *C*_U(Ⅵ)_ = 11.9 mg L^-1^, pH = 4.0 ± 0.1, and 298 K).


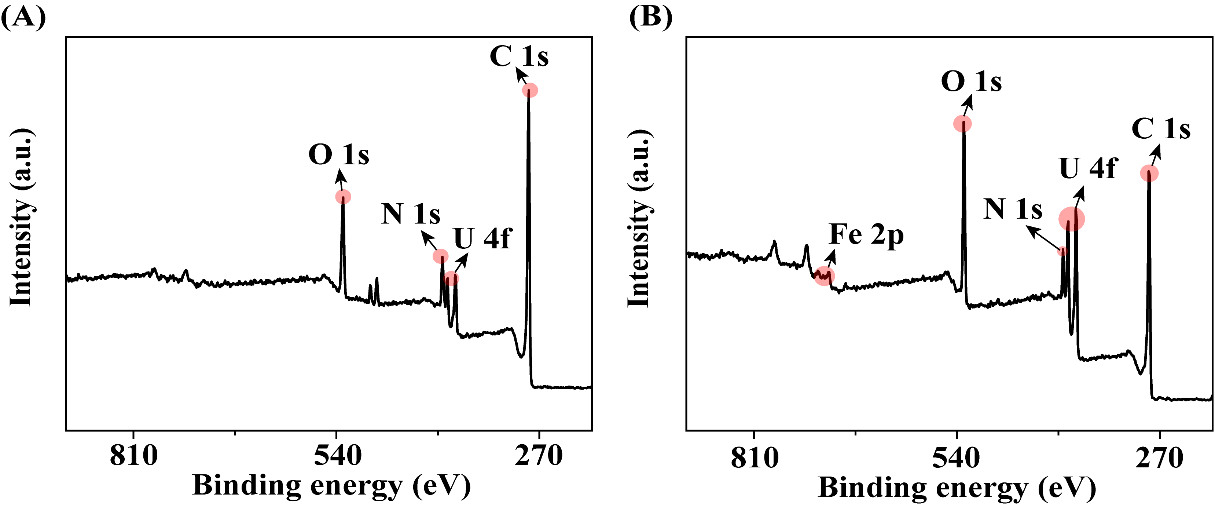


Figure S11. XPS spectra of A) PDACN after adsorption of uranyl (PDACN-U) and B) Fe@PDACN-U, containing C, N, O, U and C, N, O, Fe, U, respectively. This indicates that U(Ⅵ) was adsorbed on PDACN and Fe@PDACN.


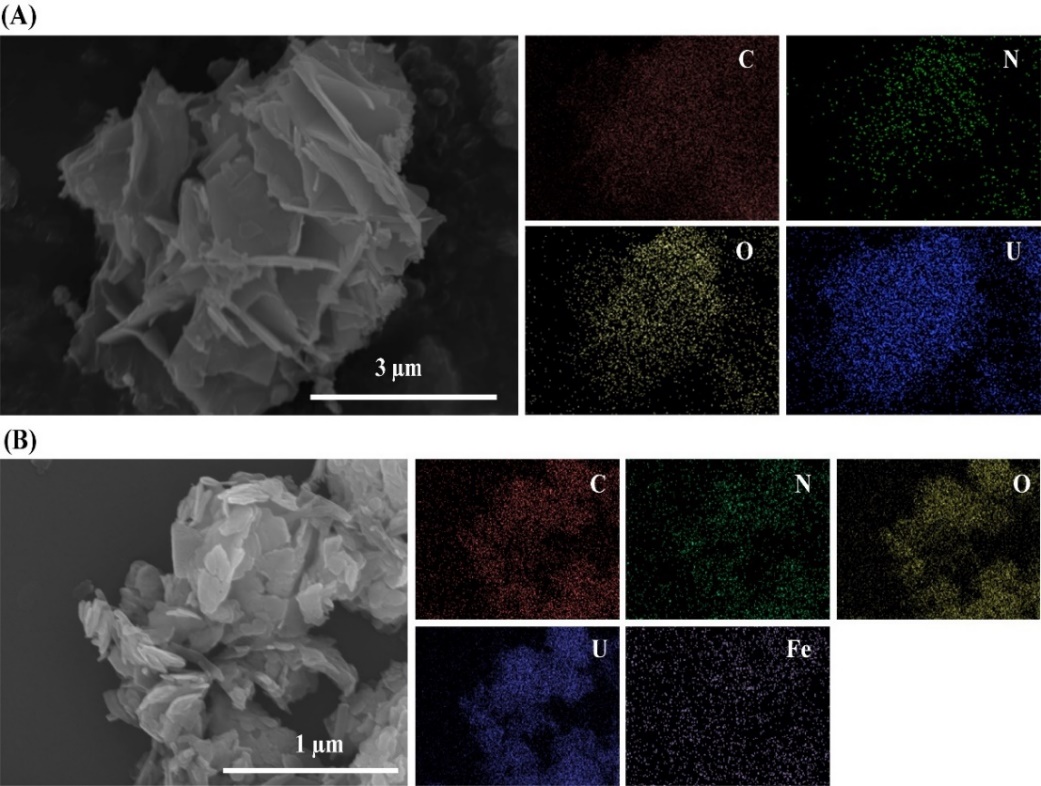


Figure S12. A) SEM images and elemental mapping images of PDACN-U. C, N, O, and U elements were evenly distributed. B) SEM images and elemental mapping images of Fe@PDACN-U. (C, N, O, Fe and U elements were evenly distributed. Both PDACN and Fe@PDACN maintained their lamellar morphology after adsorption of U(VI) when not energized.)


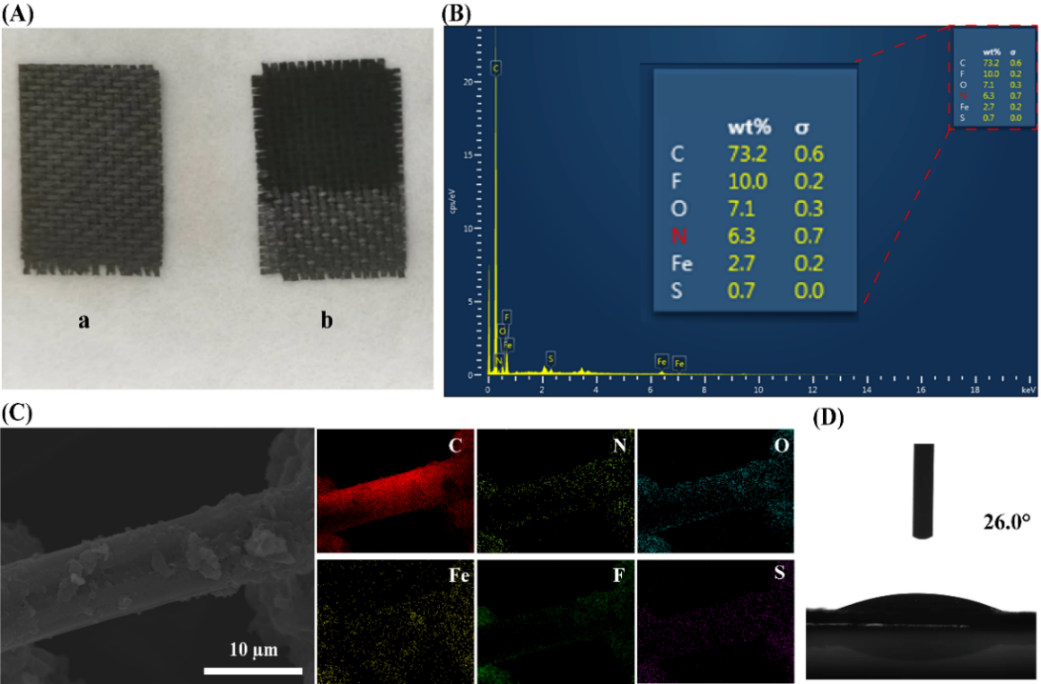


Figure S13. A) Photographs of hydrophilic conductive carbon cloth and Fe@PDACN-loaded electrodes (a: hydrophilic conductive carbon cloth, b: Fe@PDACN-loaded electrodes). B) EDS spectrum of Fe@PDACN-loaded electrodes. C) SEM images and elemental mapping images of Fe@PDACN-loaded electrodes. (C, N, O, and Fe elements were evenly distributed, indicating uniform material modification. The F and S elements were from Nafion solution. D) Contact angle for DI water on Fe@PDACN-loaded electrode.)


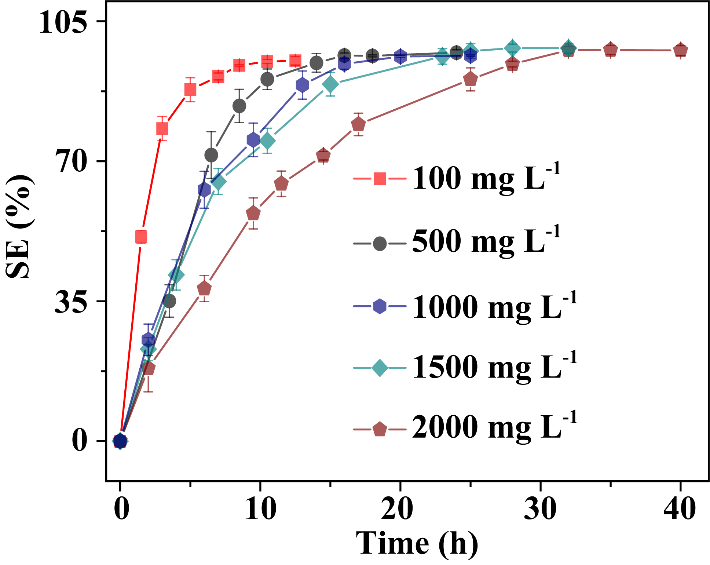


Figure S14. The efficiency of electrochemical U(Ⅵ) extraction at different initial U(Ⅵ) concentrations. Conditions: solid-liquid ratio = 0.1 g L^-1^, voltage = -1.5 V, *C*_NaCl_ = 1 mol L^-1^.


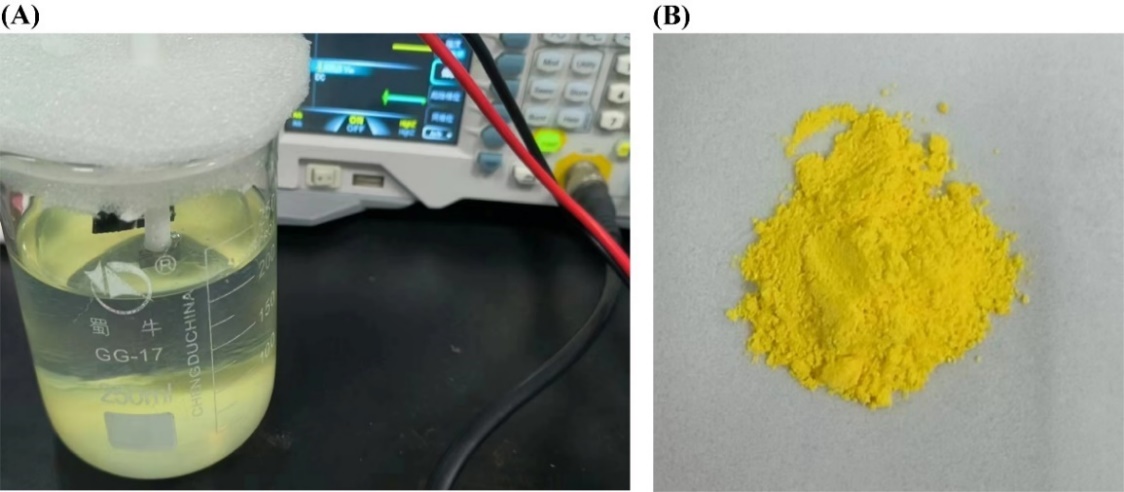


Figure S15. Photographs of A) continuous electrocatalytic extraction of uranium in simulated nuclear wastewater (Table S6) and of B) precipitates after freeze-drying. (Each extraction was carried out until the U(Ⅵ) concentration was < 1 mg L^-1^, i.e., more than 98% of uranium was recovered. The Fe@PDACN-loaded electrode did not require any treatment after the completion of each extraction and was used directly for the next electrochemical uranium extraction.)


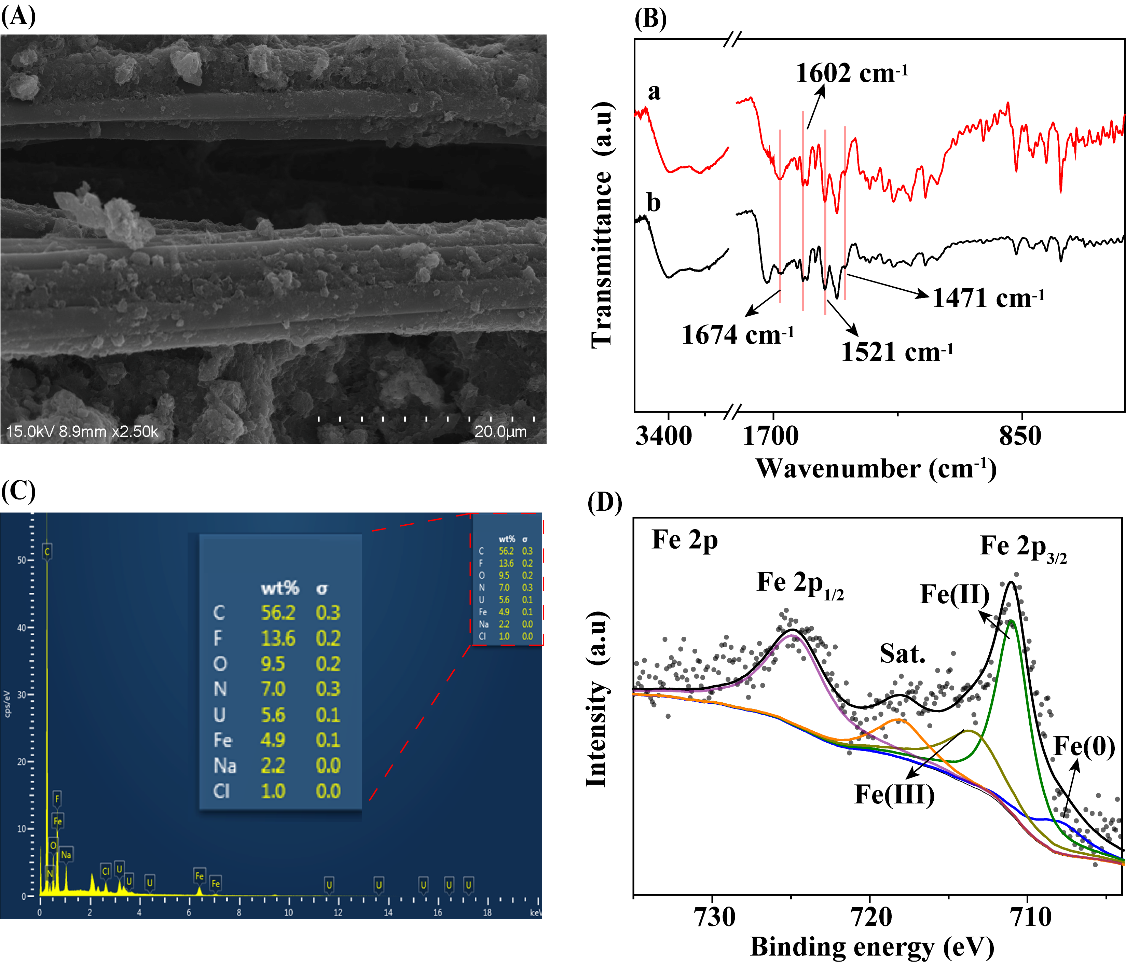


Figure S16. A) SEM image of Fe@PDACN-loaded electrode after 10 consecutive electrocatalytic uranium extraction. B) FTIR spectra of Fe@PDACN and Fe@PDACN-loaded electrode after 10 consecutive electrocatalytic uranium extraction (a: Fe@PDACN-loaded electrode after 10 consecutive electrocatalytic uranium extraction; b: fresh Fe@PDACN-loaded electrode). C) EDS spectra and D) Fe 2p XPS spectra of Fe@PDACN-loaded electrode after 10 consecutive electrocatalytic uranium extraction.


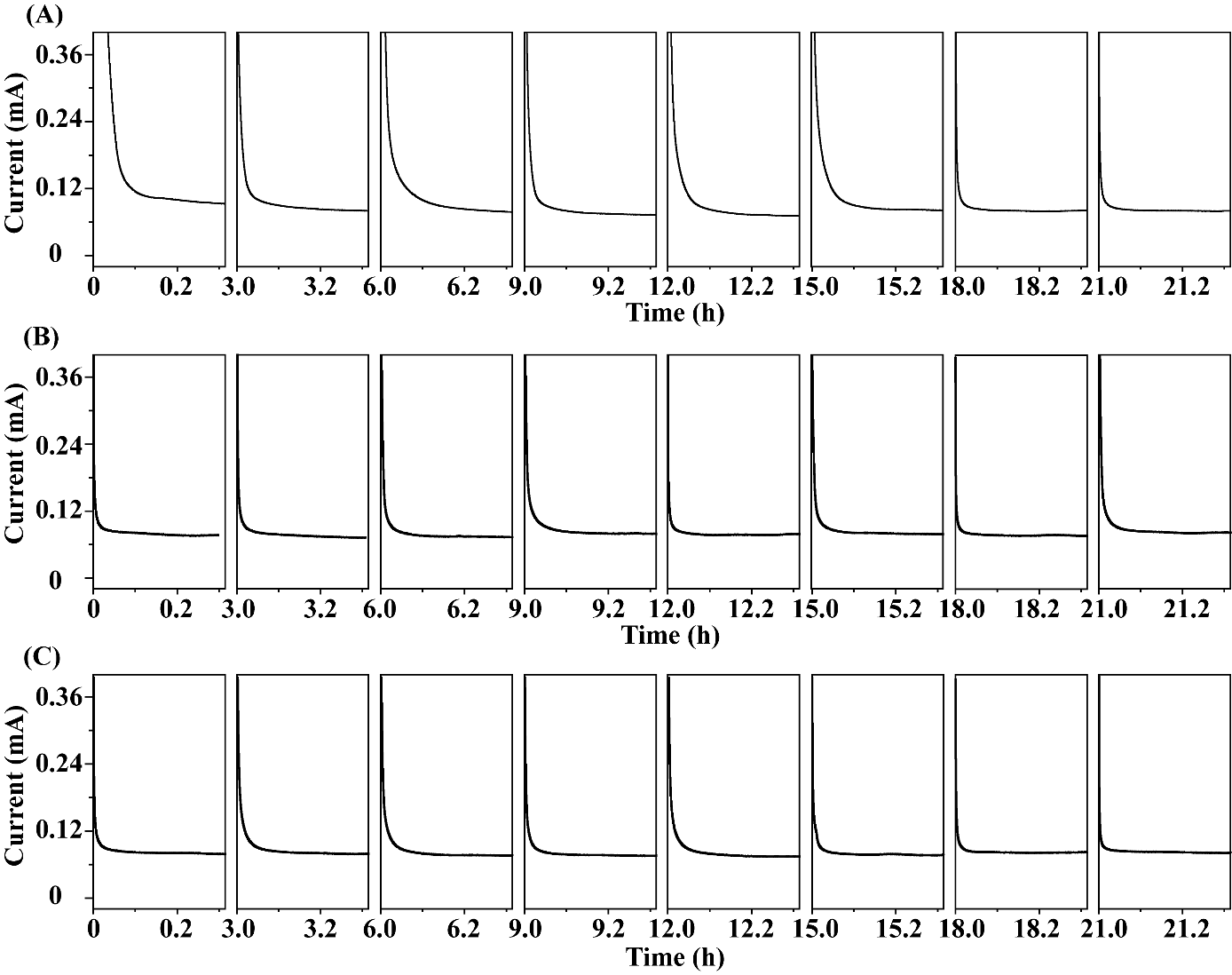


Figure S17. Current of continuous electrochemical uranium extraction: A) the first time, B) the second time and C) the third time. (The current of the first three continuous electrochemical uranium extractions was recorded in segments by chronoamperometry. Recordings were made every three hours for 1000 s to ensure current stabilization. The average current obtained by integration was 8.8 × 10^-5^ A.)


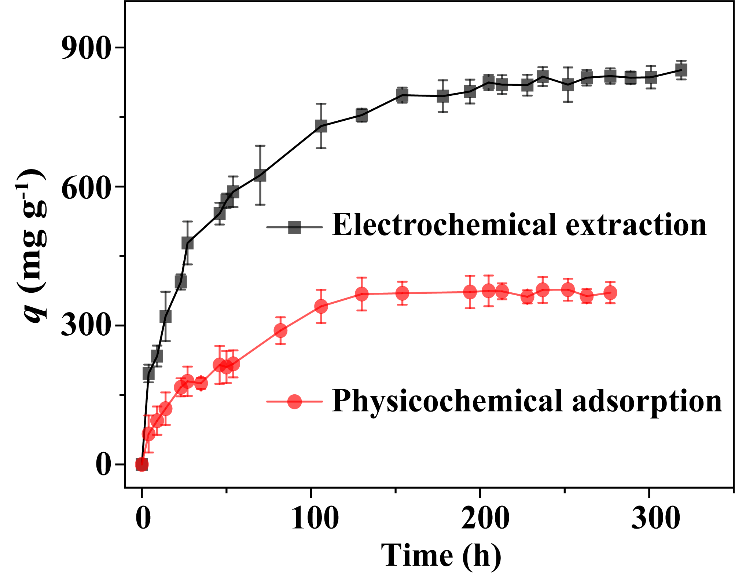


Figure S18. Efficiency of Fe@PDACN based electrochemical uranium extraction and Fe@PDACN physicochemical adsorption in simulated uranium-containing groundwater (Table S7, *C*_U(Ⅵ)_ = 1.38 mg L^-1^). Conditions: solid-liquid ratio was set at 2 mg/2000 mL, voltage = -1.5 V.


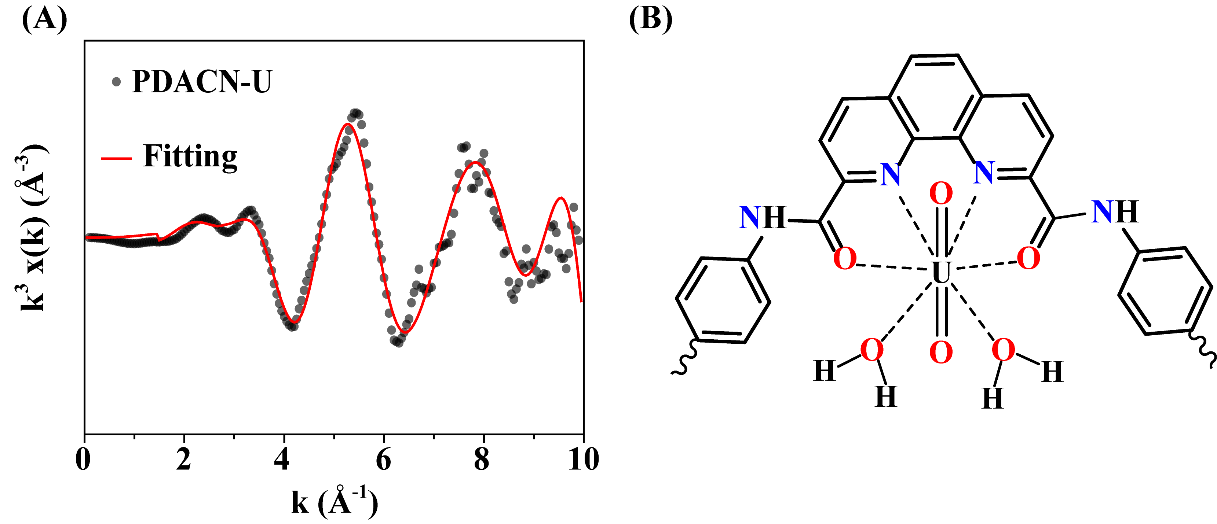


Figure S19. A) U L−edge EXAFS k−space and corresponding fitting curves for PDACN-U. B) Schematic illustration of the strong binding affinity between the PDA block and uranyl ion.


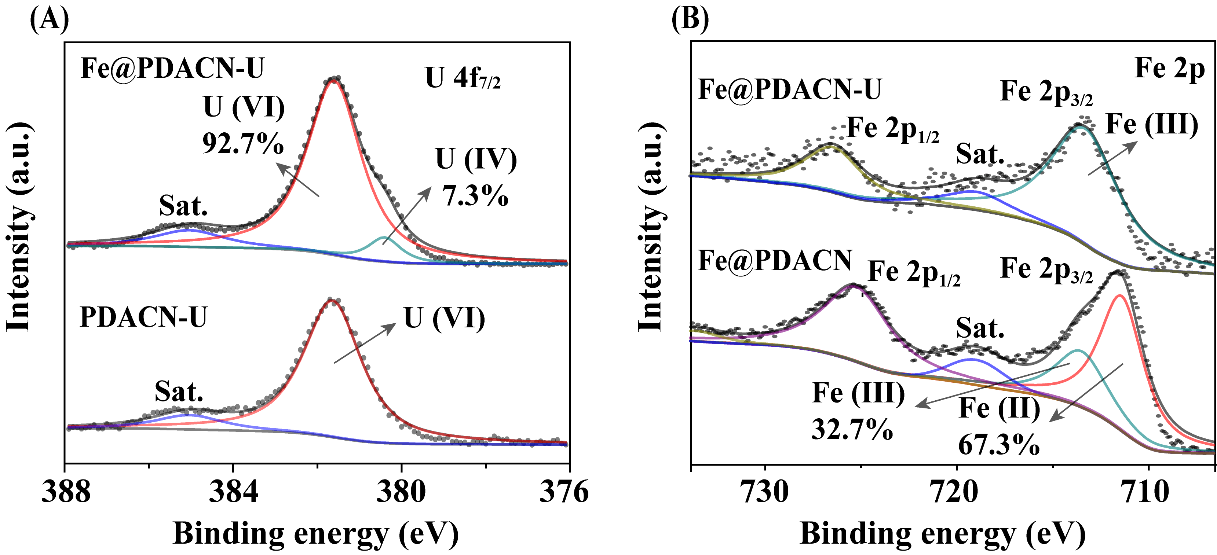


Figure S20. A) U 4f_7/2_ XPS spectra for PDACN-U and Fe@PDACN-U. B) Fe 2p XPS spectra for Fe@PDACN and Fe@PDACN-U. (The U 4f_7/2_ spectra of PDACN-U shows two characteristic peaks corresponding to U(Ⅵ) at 381.6 eV and satellites of U(Ⅵ) at 385.0 eV, with an additional peak attributed to U(Ⅳ) at 380.6 eV (7.3% of the total extracted uranium) observed in the spectrum of Fe@PDACN-U. This additional peak is associated with the reduction of partially adsorbed U(Ⅵ) to U(Ⅳ) by the Fe(Ⅱ) loaded on Fe@PDACN. The disappearance of Fe(Ⅱ) peak and the pronounced strengthening of Fe(Ⅲ) peak in the Fe 2p fine spectrum of Fe@PDACN-U, further validate the U(Ⅵ) extraction reduction mechanism using Fe@PDACN.^[53]^ The Fe 2p XPS spectrum of Fe@PDACN showed that Fe(Ⅱ) accounted for 67.3% of the total Fe content. The Fe content in Fe@PDACN determined by ICP-MS was 5.2 wt.% of the total mass, thus, the divalent Fe(Ⅱ) content in Fe@PDACN was 3.4 wt.%. The appearance of Fe(Ⅲ) in Fe@PDACN was attributed to the oxidation of partial Fe(Ⅱ) by oxygen during the preparation process. The low percentage content of Fe(Ⅱ) in Fe@PDACN leads to its limited reduction capacity for U(Ⅵ), which accounts for the similar adsorption capacity of Fe@PDACN (384.0 mg g^-1^) and PDACN (376.4 mg g^-1^) observed in physicochemical adsorption (Figure 3B and Table S4). The physicochemical adsorption mechanism involving the reduction of U(Ⅵ) to U(Ⅳ) induced by the Fe-porphyrin sites is corroborated by the above results, providing the basis for achieving high Faraday efficiency in the electrochemical extraction of U(Ⅵ).)


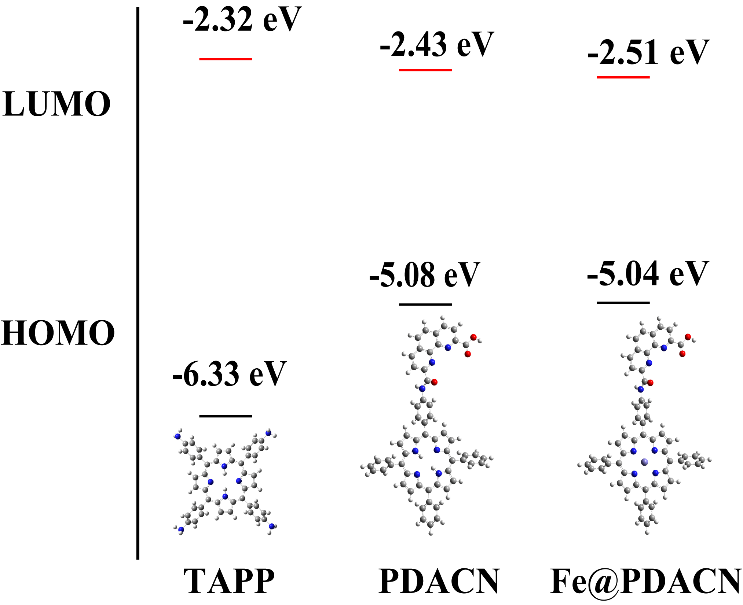


Figure S21. HOMO-LUMO of TAPP, PDACN and Fe@PDACN.


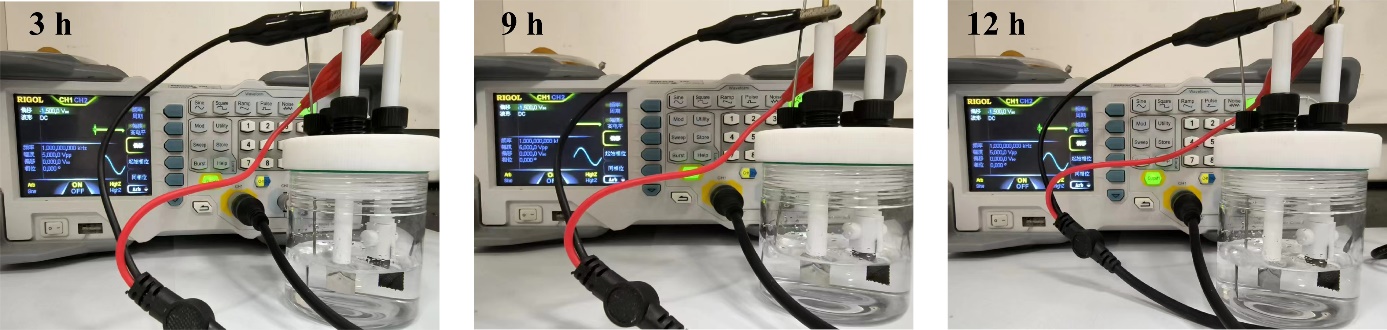


Figure S22. Photographs of electrochemical extraction in N_2_ atmosphere (3 h, 9 h and 12 h). Conditions: solid-liquid ratio = 0.1 g L^-1^, initial *C*_U(Ⅵ)_ = 100 mg L^-1^, voltage = -1.5 V, *C*_NaCl_ = 1 mol L^-1^.


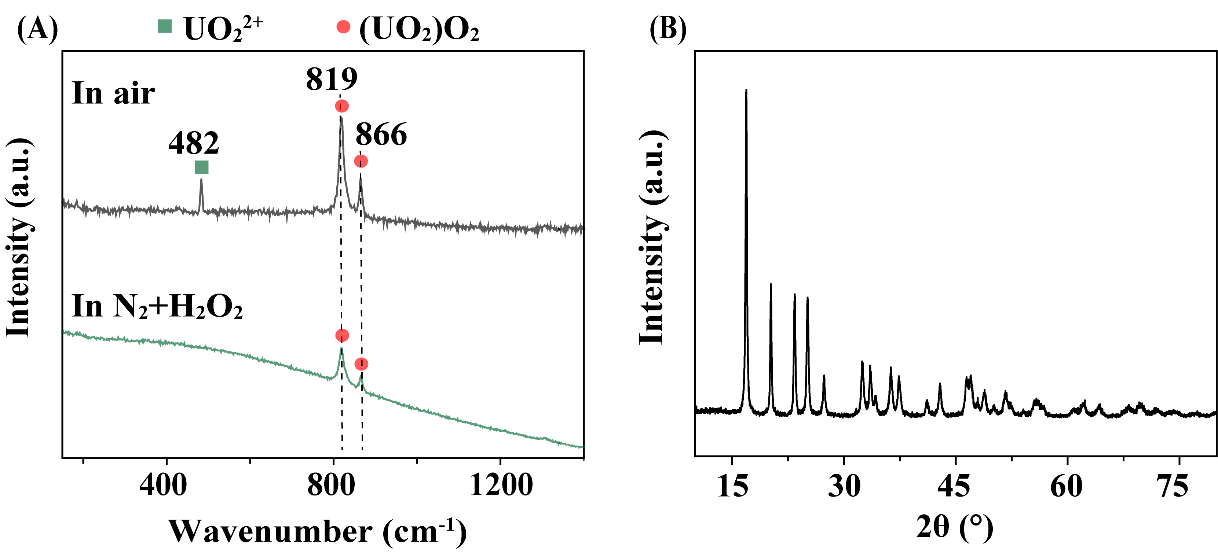


Figure S23. A) Raman spectra of the electrodeposits after electrochemical extraction in air, as well as in N_2_ containing H_2_O_2_. B) XRD pattern of the electrochemical extract obtained under N_2_ atmosphere containing H_2_O_2_, identified as (UO_2_)O_2_⋅2H_2_O.

The Raman spectra of electrodeposits collected in air or in N_2_ containing H_2_O_2_ both show peaks at 819 and 866 cm^-1^, corresponding to the species of uranium peroxide (Figure S21C).^[26, 54]^ The electrodeposits collected in test solutions containing H_2_O_2_ under N_2_ atmosphere were also identified by XRD as (UO_2_)O_2_⋅2H_2_O (Figure S21D). Combined with cyclic voltammetry analysis (Figure 6C) (i.e., the reduction peak corresponds to the reduction of U (Ⅵ) to U (V), and some U (V) disproportionation produces U (Ⅳ), resulting in the absolute peak current of the oxidation peak being lower than the reduction peak), these results support the rapid interaction between H_2_O_2_ and U(V)/U(Ⅳ) to produce the (UO_2_)O_2_⋅xH_2_O species, which is similar to previous literature^[26]^:

UO_2_^2+^ + e^-^ = UO_2_^+^ (1)

2UO_2_^+^ = UO_2_^2+^ + UO_2_ (2)

O_2_ + 2H_2_O + 2e^-^ = H_2_O_2_ + 2OH^-^ (3)

2UO_2_^+^ + 3H_2_O_2_ + (2x-2)H_2_O = 2(UO_2_)O_2_⋅xH_2_O + 2H^+^ (4)

UO_2_ + 2H_2_O_2_ + (x-2)H_2_O = (UO_2_)O_2_⋅xH_2_O (5)

## Reference

[1] a) Y. Ho, *J. Hazard. Mater.* **2006**, *136*, 681-689; b) Y. S. Ho, G. McKay, *Process Biochem.* **1999**, *34*, 451-465; c) Y. S. Ho, G. McKay, *Water Res.* **2000**, *34*, 735-742.

[2] I. Langmuir, *J. Am. Chem. Soc.* **1918**, *40*, 1361-1403.

[3] a) B. Zohuri, in *Nuclear reactor technology development and utilization* (Eds.: S. U.-D. Khan, A. Nakhabov), Woodhead Publishing, **2020**, pp. 61-120; b) H. Ma, M. Shen, Y. Tong, X. Wang, *Molecules* **2023**, *28*, 1935; c) X. Nie, Y. Zhang, Y. Jiang, N. Pan, C. Liu, J. Wang, C. Ma, X. Xia, M. Liu, H. Zhang, X. Li, F. Dong, *Sci. Total Environ.* **2022**, *831*, 154743.

[4] a) Y. Cheng, P. He, F. Dong, X. Nie, C. Ding, S. Wang, Y. Zhang, H. Liu, S. Zhou, *Chem. Eng. J.* **2019**, *367*, 198-207; b) T. Liu, J. Yuan, B. Zhang, W. Liu, L. Lin, Y. Meng, S. Yin, C. Liu, F. Luan, *Environ. Sci. Technol.* **2019**, *53*, 14612-14619.

[5] a) C. Lee, W. Yang, R. G. Parr, *Phys. Rev. B* **1988**, *37*, 785-789; b) S. Grimme, J. Antony, S. Ehrlich, H. Krieg, *J. Chem. Phys* **2010**, *132*, 154104; c) W. J. Hehre, R. Ditchfield, J. A. Pople, *J. Chem. Phys* **1972**, *56*, 2257-2261; d) D. Andrae, U. Häußermann, M. Dolg, H. Stoll, H. Preuß, *Theor. Chim. Acta* **1990**, *77*, 123-141.

[6] a) Y. Zhao, D. G. Truhlar, *Theor. Chem. Acc.* **2008**, *120*, 215-241; b) M. Ortiz, J. Campos, *J. Chem. Phys* **1980**, *72*, 5635-5638; c) T. Clark, J. Chandrasekhar, G. W. Spitznagel, P. V. R. Schleyer, *J. Comput. Chem.* **1983**, *4*, 294-301; d) H.F. Lu, H.F. Chen, C.L. Kao, I. Chao, H.Y. Chen, *Phys. Chem. Chem. Phys.* **2018**, *20*, 22890-22901.

[7] C. Zhang, C. Kong, P. G. Tratnyek, C. Qin, *Environ. Sci. Technol.* **2022**, *56*, 4367-4376.

[8] M. Huang, L. Xie, Y. Wang, X. Feng, J. Gao, Z. Lou, Y. Xiong, *Environ. Pollut.* **2023**, *316*, 120550.

[9] Y. Xie, C. Chen, X. Ren, X. Tan, G. Song, D. Chen, A. Alsaedi, T. Hayat, *J. Colloid Interface Sci.* **2019**, *550*, 117-127.

[10] D. Zhao, Y. Wang, S. Zhao, M. Wakeel, Z. Wang, R. S. Shaikh, T. Hayat, C. Chen, *Environ. Pollut.* **2019**, *251*, 547-554.

[11] S. Yang, Y. Cao, T. Wang, S. Cai, M. Xu, W. Lu, D. Hua, *Environ. Res.* **2020**, *183*, 109214.

[12] D. Wen, C. Xie, Z. Dong, M. Zhang, M. Zhai, L. Zhao, *Sep. Purif. Technol.* **2022**, *301*, 121969.

[13] J. Zhu, L. Zhao, D. Song, J. Yu, Q. Liu, J. Liu, R. Chen, G. Sun, J. Wang, *Desalination* **2022**, *540*, 115993.

[14] J. Bai, S. Li, X. Ma, H. Yan, S. Su, S. Wang, J. Wang, *Microporous Mesoporous Mater.* **2022**, *331*, 111647.

[15] S. Yu, J. Wang, S. Song, K. Sun, J. Li, X. Wang, Z. Chen, X. Wang, *Sci. China Chem.* **2017**, *60*, 415-422.

[16] J. Das, A. Rawat, L. Singh, A. Maiti, A. Bhatnagar, P. Mohanty, *ACS Appl. Eng. Mater.* **2023**, *1*, 2004-2017.

[17] V. S. Vaddanam, A. Pamarthi, S. Sengupta, M. Sahoo, S. K. Gupta, S. Balija, G. Gopakumar, C. V. S. Brahmananda Rao, A. Suresh, S. N. Jha, S. Nagarajan, *ACS Appl. Nano Mater.* **2023**, *6*, 8222-8237.

[18] R. Liu, Q. Wan, Y. Yu, X. Zhang, L. Liu, H. Wang, C. Yue, *J. Water Process Eng.* **2023**, *53*, 103659.

[19] H. Zhang, S. Wang, J. Yu, Z. Li, J. Lan, L. Zheng, S. Liu, L. Yuan, T. Xiu, J. Wang, X. Wang, W. Shi, *Chem. Eng. J.* **2023**, *463*, 142408.

[20] M. Xu, L. Zhou, L. Zhang, S. Zhang, F. Chen, R. Zhou, D. Hua, *ACS Appl. Mater. Interfaces* **2022**, *14*, 9408-9417.

[21] Z. Wang, R. Ma, Q. Meng, Y. Yang, X. Ma, X. Ruan, Y. Yuan, G. Zhu, *J. Am. Chem. Soc.* **2021**, *143*, 14523-14529.

[22] Q. Liu, N. Wang, B. Xie, D. Xiao, *Sep. Purif. Technol.* **2023**, *308*, 122866.

[23] J. Ao, H. Zhang, X. Xu, F. Yao, L. Ma, L. Zhang, B. Ye, Q. Li, L. Xu, H. Ma, *RSC Adv.* **2019**, *9*, 28588-28597.

[24] J. Gan, L. Zhang, Q. Wang, Q. Xin, Y. Xiong, E. Hu, Z. Lei, H. Wang, H. Wang, *Int. J. Biol. Macromol.* **2023**, *238*, 124074.

[25] H. Yang, X. Liu, M. Hao, Y. Xie, X. Wang, H. Tian, G. I. N. Waterhouse, P. E. Kruger, S. G. Telfer, S. Ma, *Adv. Mater.* **2021**, *33*, 2106621.

[26] C. Liu, P. Hsu, J. Xie, J. Zhao, T. Wu, H. Wang, W. Liu, J. Zhang, S. Chu, Y. Cui, *Nat. Energy* **2017**, *2*, 17007.

[27] Z. Gao, S. Chen, H. Ding, Y. Song, Z. Li, H. Wang, H. Wu, H. Li, Y. Su, *Inorg. Chem. Commun.* **2022**, *146*, 110134.

[28] R. Cao, J. Zhang, D. Wang, F. Sun, N. Li, J. Li, *Chem. Eng. J.* **2023**, *461*, 142080.

[29] Y. Liao, R. Lei, X. Weng, C. Yan, J. Fu, G. Wei, C. Zhang, M. Wang, H. Wang, *J. Hazard. Mater.* **2023**, *442*, 130054.

[30] D. Wang, J. Zhou, Y. Zhang, J. Zhang, J. Liang, J. Zhang, J. Li, *Chem. Eng. J.* **2023**, *463*, 142413.

[31] H. Yu, L. Zhou, Z. Li, Y. Liu, X. Ao, J. Ouyang, Z. Le, Z. Liu, A. A. Adesina, *Sep. Purif. Technol.* **2022**, *302*, 122169.

[32] C. Wang, A. S. Helal, Z. Wang, J. Zhou, X. Yao, Z. Shi, Y. Ren, J. Lee, J. Chang, B. Fugetsu, J. Li, *Adv. Mater.* **2021**, *33*, 2102633.

[33] Y. Ye, B. Fan, Z. Qin, X. Tang, Y. Feng, M. Lv, S. Miao, H. Li, Y. Chen, F. Chen, Y. Wang, *J. Hazard. Mater.* **2022**, *432*, 128723.

[34] X. Liu, Y. Xie, M. Hao, Z. Chen, H. Yang, G. I. N. Waterhouse, S. Ma, X. Wang, *Adv. Sci.* **2022**, *9*, 2201735.

[35] M. Huang, L. Xie, Y. Wang, H. He, H. Yu, J. Cui, X. Feng, Z. Lou, Y. Xiong, *Chem. Eng. J.* **2023**, *457*, 141221.

[36] M. Pan, C. Cui, W. Tang, Z. Guo, D. Zhang, X. Xu, J. Li, *Sep. Purif. Technol.* **2022**, *281*, 119843.

[37] X. Tang, Y. Liu, M. Liu, H. Chen, P. Huang, H. Ruan, Y. Zheng, F. Yang, R. He, W. Zhu, *Nanoscale* **2022**, *14*, 6285-6290.

[38] H. Ye, T. Li, Y. Huang, J. Jin, J. Fei, M. Wu, J. Yao, *Chem. Eng. J.* **2023**, *451*, 138615.

[39] J. Li, C. Jiao, Y. Lin, Y. Li, Z. Qian, H. Liu, T. Chen, Y. Liu, R. He, W. Zhu, *Appl. Catal., B* **2024**, *347*, 123770.

[40] Z. Wang, Q. Meng, R. Ma, Z. Wang, Y. Yang, H. Sha, X. Ma, X. Ruan, X. Zou, Y. Yuan, G. Zhu, *Chem* **2020**, *6*, 1683-1691.

[41] H. Li, N. He, C. Cheng, H. Dong, J. Wen, X. Wang, *Chem. Eng. J.* **2020**, *388*, 124273.

[42] S. Shi, Y. Qian, P. Mei, Y. Yuan, N. Jia, M. Dong, J. Fan, Z. Guo, N. Wang, *Nano Energy* **2020**, *71*, 104629.

[43] W. Sun, L. Feng, J. Zhang, K. Lin, H. Wang, B. Yan, T. Feng, M. Cao, T. Liu, Y. Yuan, N. Wang, *Adv. Sci.* **2022**, *9*, 2105008.

[44] T. Liu, R. Zhang, M. Chen, Y. Liu, Z. Xie, S. Tang, Y. Yuan, N. Wang, *Adv. Funct. Mater.* **2022**, *32*, 2111049.

[45] L. Feng, H. Wang, T. Feng, B. Yan, Q. Yu, J. Zhang, Z. Guo, Y. Yuan, C. Ma, T. Liu, N. Wang, *Angew. Chem. Int. Ed.* **2022**, *61*, e202101015.

[46] X. Xu, L. Xu, J. Ao, Y. Liang, C. Li, Y. Wang, C. Huang, F. Ye, Q. Li, X. Guo, J. Li, H. Wang, S. Ma, H. Ma, *J. Mater. Chem. A* **2020**, *8*, 22032-22044.

[47] B. Yan, C. Ma, J. Gao, Y. Yuan, N. Wang, *Adv. Mater.* **2020**, *32*, 1906615.

[48] W. Cui, F. Li, R. Xu, C. Zhang, X. Chen, R. Yan, R. Liang, J. Qiu, *Angew. Chem. Int. Ed.* **2020**, *59*, 17684-17690.

[49] L. Chen, Z. Bai, L. Zhu, L. Zhang, Y. Cai, Y. Li, W. Liu, Y. Wang, L. Chen, J. Diwu, J. Wang, Z. Chai, S. Wang, *ACS Appl. Mater. Interfaces* **2017**, *9*, 32446-32451.

[50] X. Xu, H. Zhang, J. Ao, L. Xu, X. Liu, X. Guo, J. Li, L. Zhang, Q. Li, X. Zhao, B. Ye, D. Wang, F. Shen, H. Ma, *Energy Environ. Sci.* **2019**, *12*, 1979-1988.

[51] S. Yano, K. Sato, J. Suzuki, H. Imai, Y. Oaki, *Commun. Chem* **2019**, *2*, 97.

[52] a) C.L. Xiao, C.Z. Wang, L.Y. Yuan, B. Li, H. He, S. Wang, Y.L. Zhao, Z.F. Chai, W.Q. Shi, *Inorg. Chem.* **2014**, *53*, 1712-1720; b) C.L. Xiao, Q.Y. Wu, C.Z. Wang, Y.L. Zhao, Z.F. Chai, W.Q. Shi, *Inorg. Chem.* **2014**, *53*, 10846-10853.

[53] J. Peng, H. Zhou, W. Liu, Z. Ao, H. Ji, Y. Liu, S. Su, G. Yao, B. Lai, *Chem. Eng. J.* **2020**, *397*, 125387.

[54] D. Manara, B. Renker, *J. Nucl. Mater.* **2003**, *321*, 233-237.
